# The Patterns and Causes of Dermatitis in Terrestrial and Semi-Aquatic Mammalian Wildlife

**DOI:** 10.3390/ani11061691

**Published:** 2021-06-06

**Authors:** Elise M. Ringwaldt, Barry W. Brook, Scott Carver, Jessie C. Buettel

**Affiliations:** School of Natural Sciences, University of Tasmania, Hobart, TAS 7000, Australia; Barry.Brook@utas.edu.au (B.W.B.); Scott.Carver@utas.edu.au (S.C.); Jessie.Buettel@utas.edu.au (J.C.B.)

**Keywords:** captivity, dermatitis, dermatophilosis, exudative dermatitis, free-living, mammal, threatened species, wildlife

## Abstract

**Simple Summary:**

Dermatitis is recognized to manifest from a variety of causes in humans and animals, but these have never been synthesized for wildlife. We document the causes and investigate the magnitude of skin diseases and disorders which manifest as, and are described as, dermatitis in the published literature. Our aim was to identify the major causal agents in the development of dermatitis, and if certain mammalian wildlife groups or captivity status was a precursor for the development of dermatitis. The most common causes of dermatitis identified were mites, bacteria, and fungus, and were more frequently reported for wildlife species within Carnivora and Artiodactyla. Furthermore, associated with genetic and behavioral variables, some threatened species were more likely to exhibit dermatitis in captivity. This study provides insight into wild mammalian species that may be more susceptible to dermatological diseases and discusses the patterns of causes within wild mammals.

**Abstract:**

Causative disease and stress agents which manifest as dermatitis in mammals have varying effects on individual animals, from benign irritation and inflammation, to causing morbidity and even mortality. Bacteria, viruses and ectoparasites are all potential causes of dermatitis, and it can be exacerbated by various environmental, genetic and social factors. Furthermore, it is uncertain whether dermatitis is more likely to manifest in already-vulnerable wildlife species. Here, we systematically review the literature for reports of dermatitis in terrestrial and semi-aquatic wild mammalian species, with the goal of determining the biogeographical scale of dermatitis reports, the causes of dermatitis, and whether manifestation of dermatitis is reported more commonly in certain wildlife species or their captivity status (i.e., free-living, in captivity or in a laboratory). We reveal biases in the reporting of dermatitis by a biogeographic realm, with 55% of cases reported in the Nearctic, and towards particular orders of mammals, namely Artiodactyla and Carnivora. Overall, free-living wildlife is almost twice as likely to be reported as having dermatitis than individuals in captivity and six times more likely than individuals in laboratories, which we interpret as owing to exposure to a broader spectrum of parasites in free-ranging individuals, and potential reporting bias in captive individuals. Notably, dermatitis was reported in 23 threatened species, with some species more likely than others to be reported exhibiting clinical signs of dermatitis resulting from underlying health problems. We also find that threatened species are more likely to be reported as having dermatitis in captivity, particularly outside of their endemic home range. This review highlights diverse patterns of dermatological disease causes in captive and free-ranging wildlife, conditions under which they are more likely to be documented, and the need for cross-disciplinary research to ascertain (and so better manage) the varied causes.

## 1. Introduction

Dermatitis is a general term for describing different forms of skin irritation and inflammation in humans and animals, with some causes of dermatitis being zoonotic and transmissible between people, domestic or captive animals and wildlife [1,2]. Dermatitis in humans and animals has been associated with reduced quality of life [3,4] and reports of dermatitis in threatened species indicates that skin diseases can also be a threat to persistence [5]. Dermatitis is the term for inflammation of the skin, which can have many different possible causes [2]. The development of clinical signs of dermatitis can be due to health issues such as infectious agents, ectoparasites, environmental irritants, or unknown [6,7]. Research into the cause and treatment of dermatitis has focused on either human health (e.g., [8,9]), or domestic animals and livestock (e.g., [10]). For some wildlife species, including many of conservation concern, the etiological agent or mechanisms behind dermatitis origins are unclear (e.g., [11,12]). The etiological agents which can trigger dermatitis may cause different effects in individuals; however, the question of whether there are patterns to the formation of dermatitis clinical signs for diseases or irritants across wildlife has yet to be determined.

Clinically, there are different types of dermatitis that can be detected by physical examination, sampling techniques (e.g., skin swabbing, skin scrapings, UV light), or may require histological examination of tissues [13]. Following detection of dermal lesions, a common challenge is identifying the causal agent(s). This is because the causes of dermatitis are variable and can also be species specific, for instance, grey squirrels (*Sciurus carolinensis*) do not display clinical signs to the parapoxvirus but spread it to red squirrels (*Sciurus vulgaris*) where it, by contrast, causes exudative dermatological lesions [12,14]. Even within the same species, dermatitis can manifest in a range of ways, from benign, moderate (e.g., hair loss), to severe (e.g., ulcerative pustules and necrosis) and mortality causing [5,15]. Benign dermatitis lesions can also lead to secondary infections through self-trauma or exposure to opportunistic pathogens [16,17,18].

Historically, research has primarily focused on wildlife exhibiting dermatitis acting as reservoirs for the pathogen, potentially spilling over into livestock and causing economic losses in agriculture animals, or transmission to humans due to proximity [1,2,10,19]. However, more recently, due to the increasing recognition of the role of infectious disease in the modern biodiversity crisis, some skin diseases, with dermatitis as an important clinical sign, such as Sarcoptic mange and White Nose Syndrome, have been viewed as an additional conservation pressure on already-threatened species [20,21,22,23]. Furthermore, it is unclear if there are generalities to the onset and progression of the clinical signs of dermatitis as a manifestation of skin disease across taxa.

The occurrence of dermatitis in wildlife species is global, and, from an animal ethics perspective, morbidity and mortality from secondary skin infections can be an incontrovertible wildlife management issue [10,24]. Dermatitis has been reported in mammalian wildlife which are already of conservation concern (e.g., [25,26,27]) and in some cases animals have died with severe dermatitis or secondary infections [28]. Within the last 10 years, the specific term ‘exudative dermatitis’ has been used as a common descriptive symptom in wildlife reports (e.g., [12,29]) and is a type of emerging dermatitis in some Australian wildlife [30,31]. Therefore, investigating whether specific types of mammalian wildlife are more likely to be reported as showing dermatitis as a manifestation of disease, and due to which causal agent, may aid in understanding skin disease risks.

In this review, we aim to consolidate the literature of terrestrial and semi-aquatic mammalian wildlife that are reported to exhibit dermatitis: firstly, to explore whether reports of dermatitis are more likely for types of etiological agents; and, secondly, whether documentation of dermatitis is similar across mammalian wildlife groups or reported more often in some orders. Additionally, we also identify biases in the reporting of dermatitis, and investigate threatened species reported as suffering from dermatitis as an added pressure for species already subjected to environmental and anthropogenic threats. We attempt to be exhaustive in our search of published reports of dermatitis in the literature, and results herein broadly align with general perceptions across a range of texts and sources. We recognize not all cases fitting the definition are always explicitly termed ‘dermatitis’ in wildlife, and thus do not claim this review to cover every possible study encompassing dermatitis symptoms (which we discuss), but we have reasons to be confident that this review captures general patterns and is thus an appropriate synthesis contributing a general understanding of this condition in wildlife.

## 2. Database Search and Literature Screening

**Figure 1:**
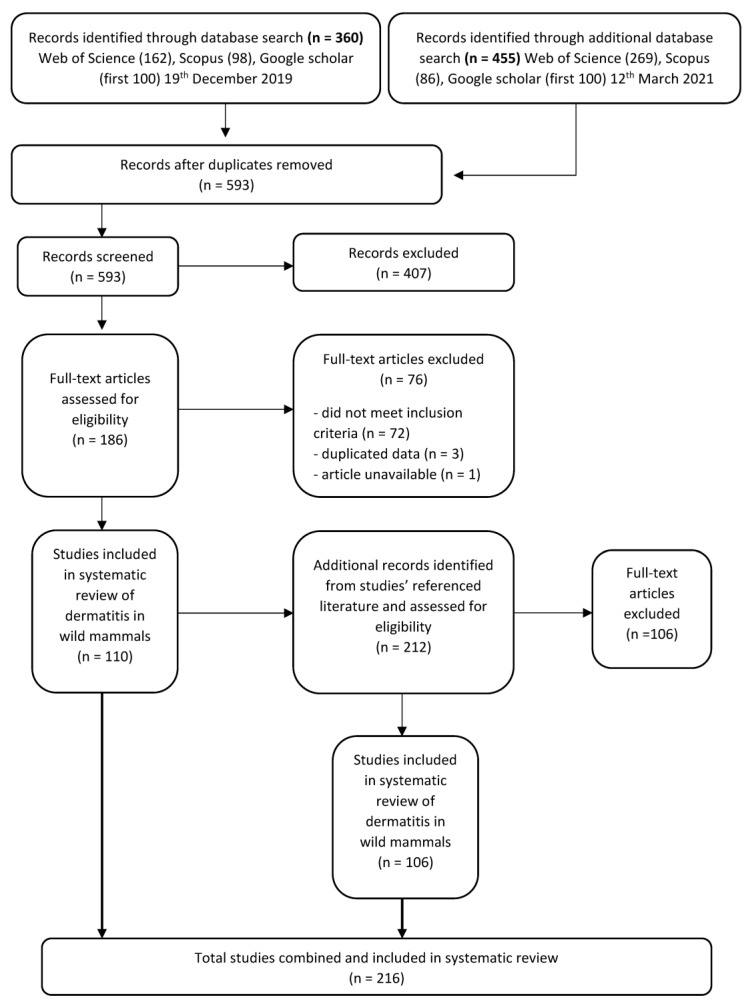
Protocol for screening articles after data base search of “dermatitis” OR “exudative dermatitis” AND “wildlife”. Each step shows the number of papers included or excluded for review.

To identify the relevant literature, we searched three databases in December 2019 and additionally in March 2021: Web of Science, Scopus and Google Scholar using the search string in the topic of: title, abstract and key words, ‘“dermatitis” OR “exudative dermatitis” AND “wildlife”’ (Search terms S1). We imported all records from Web of Science and Scopus, and the first-100 papers (ranked-by-relevance) were exported from Google Scholar for screening to reduce disparate articles (Figure 1). We restricted our literature review to peer-reviewed publications—as the most robust and defendable source of reports—available in these most widely used and publicly available databases to researchers. We acknowledge that this will preclude some non-journal literature (e.g., annual reports, conference proceedings, books) available in less widely available databases, such as CAB, which may contain additional reports that could be considered in future analyses.

After duplicate articles were removed, articles were first screened by title and abstract for relevance to dermatitis and wildlife and those not meeting the inclusion criteria were removed (Figure 1). We then assessed the full text of the filtered list of articles to identify records that fit our eligibility criteria. We only included articles which described anywhere in text, signs of histopathological or superficial dermatitis affecting the mammal in question. To ensure comprehensiveness of the literature search, we also screened the bibliographies and reference lists from the final full-text articles (Figure 1).

### 2.1. Inclusion and Exclusion Criteria

All articles were included in this review if they focused on wild (terrestrial or semi-aquatic) mammals that reported physical lesions that were diagnosed as dermatitis (regardless of the cause). Articles in this review only included records of mammals exhibiting dermatological clinical signs, and, therefore, manuscripts which focused exclusively on serological surveys of pathogens/antibodies which may cause dermatitis were excluded. Articles were also excluded if the focus of the research was a review, conference proceedings, or book review. After the screening process, articles were also excluded if they only used data from a previously published study.

### 2.2. Data Extraction

For each article, we extracted numeric, categorical, and written information about the manuscript, mammal species exhibiting dermatitis, and the agent causing dermatitis (Table 1; Table S2).

For articles with multiple species, we recorded data for all individuals exhibiting signs of dermatitis. When more than one species of mammal was studied, we separated each species record and, therefore, some papers are represented more than once for the species-specific analyses. We included data on the animal’s captivity status, whether it was free-living, captive (i.e., contained inside a boarder such as a zoo or wildlife park or reserve), or laboratory (i.e., a wild species to be used for laboratory experiments). To determine the total number of dermatitis cases, for each paper, we split the data by species reported (*n* = 244 individual species cases of dermatitis), then by the cause of dermatitis (*n* = 253 differential cases of dermatitis), and, finally, by the individual mammals’ captivity status (*n* = 257 cases). We also recorded the diagnosed type of dermatitis for each species in the article (e.g., exudative dermatitis, ulcerative dermatitis) as well as whether the dermatitis had a definitive causal agent identified (e.g., virus, bacteria, fungus, ectoparasite). If the causal agent of the dermatitis was not found, we classed the cause as ‘unknown’.

The country for each dermatitis case was documented and each was aggregated into their corresponding biogeographic realms (also known as ecozones). The World Wildlife Fund classification of biogeographical realms [32,33] has eight ecozones, being the broadest biogeographic division of all terrestrial ecosystems on Earth. These are delineated by broad geographical features, such as oceans and mountain ranges which have, over time, segregated evolving organisms. Therefore, biogeographic realms are based on zoogeography evolutionary histories, ecoregions, and phytogeography, among many other classification systems [33].

Species were updated to their current scientific name if it changed since the article was published (e.g., *Thalarctos maritimus* to *Ursus maritimus*), or, if a subspecies was recorded, we aggregated under the species name. We grouped species to higher taxa for further analyses based on the types of dermatitis affecting similar wildlife, as follows: Artiodactyla, even-toed ungulates (24 species); we split Carnivora into two groups, semi-aquatic Carnivora (represented by five species of sea lions and seals from the family Otariidae and Phocidae), and terrestrial Carnivora (20 species including felids, canids, and ursids); Diprotodontia, marsupial mammals (14 species); Primates, non-human eutherian mammals (nine species); and Rodentia, rodents (11 species). Five other orders had 10 or less dermatitis diagnoses and fewer than five species within the grouping. Their individual syntheses are produced in supplementary tables (Table S3): Didelphimorphia, represented by two species of opossum; Eulipotyphla, represented by four species of hedgehog; Lagomorpha, hare and rabbit (four species); Perissodactyla, rhinoceros (three species); *Chiroptera*, represented by four species of bat from the family Vespertilionidae. Finally, four unique species did not belong to any former (broader) order noted above: *Elephas maximus* (Asian elephant), *Ornithorhynchus anatinus* (platypus), *Procavia capensis* (rock hyrax), and *Zaedyus pichiy* (pichi) (Table S3).

We used the International Union for Conservation of Nature’s Red List (hereafter IUCN) (2021) to rank each species via their threat level [34]: Not Evaluated, Data Deficient, Least Concern, Near Threatened, Vulnerable, Endangered, Critically Endangered, Extinct in the Wild and Extinct. We also used this as a tool to rank the species with reported dermatitis cases by global conservation status.

## 3. Overall Causes, Captivity Status, and Geographic Spread of Reported Dermatitis

Dermatitis in wildlife was reported in 216 papers (1925–2021), totaling 257 dermatitis cases reported for terrestrial and semi-aquatic wildlife mammals (Table S2). There were 17 definitive causes (and another group for unknown) of dermatitis in wildlife, representing 76.4% of all cases (Table 2). The highest single proportion of dermatitis cases were of an unknown cause (23.7%, *n* = 61), followed closely by mites (21.4%, *n* = 55) and then both bacteria (16.3%, *n* = 42) and viruses (16.3%, *n* = 42) (Table 2). Mites as the causative agent were found across the highest number of unique wildlife species (34 species), followed by bacteria (29 species) and then both viruses and fugus (15 species) (Table 2).

**Figure 2:**
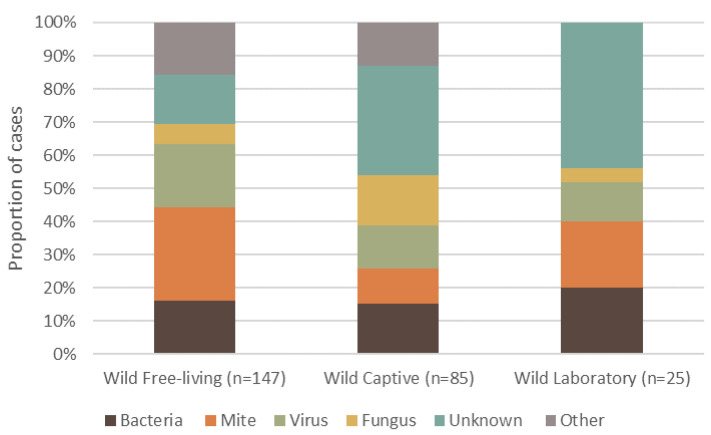
Proportion of the top five causes of dermatitis reported in terrestrial mammalian wildlife, summarized into each captive or free-ranging group. Captivity status consists of wild free-living, wild-captive and wild-laboratory. The percentage of definitive dermatitis causes reported in wildlife is shown for each group.

Dermatitis was reported in 108 different mammal species, totaling 4118 individuals. Wild, free-living mammals had a higher frequency of reported dermatitis cases (57.2%, *n* = 147, 69 species), compared to wild animals that were brought into or born in captivity (33.07%, *n* = 85, 51 species), and those under laboratory conditions (9.73%, *n* = 25, 12 species) (Figure 2). The highest proportion of reported cases of dermatitis for free-living wildlife was caused by mites (Figure 2). By contrast, unknown causes of dermatitis in captive wildlife had the highest proportion of cases. Interestingly, wildlife from a laboratory setting had no ‘other’ causes, and the primary cause of dermatitis was unknown (Figure 2).

**Figure 3:**
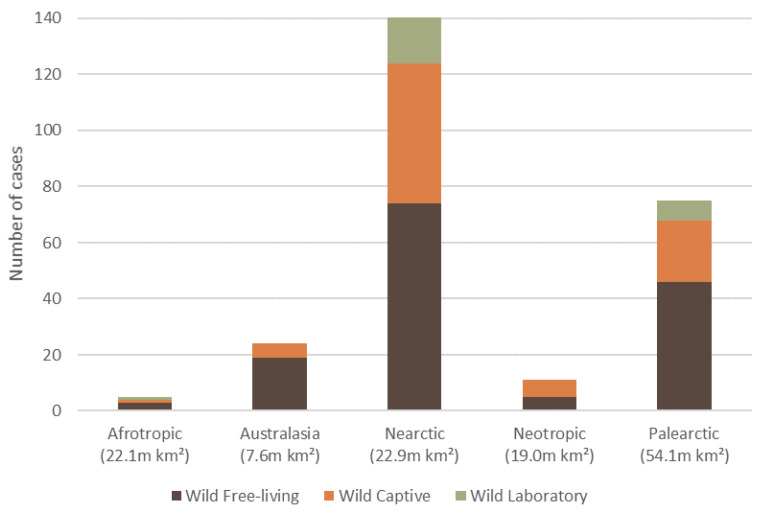
The total number of reported dermatitis cases split into captivity status (wild free-living, wild captive and wild laboratory), for five of the six biogeographical realms (ecozone) where mammalian wildlife dermatitis cases were reported in the literature, with an ecozone area (million km^2^). There was one wild captive case recorded for the Indomalaya (7.5 m km^2^) ecozone, which is not displayed.

The 257 wildlife dermatitis cases spanned six biogeographical realms (hereafter ‘ecozone’). The Nearctic had the highest number of reported cases of dermatitis (54.9%, *n* = 141, 59 species), followed by Palearctic (29.2%, *n* = 75, 37 species), Australasia (9.3%, *n* = 24, 18 species), Neotropics (4.3%, *n* = 11, 9 species), Afrotropics (1.9%, *n* = 5, 4 species), and Indomalaya (0.4%, *n* = 1, 1 species) (Figure 3).

## 4. Etiological Agents Responsible for the Causes of Dermatitis across Mammalian Wildlife Species

There were over 60 causal agents of dermatitis reported in the 108 mammalian wildlife species (Table S4). Due to diagnostic features, identification of some pathogens could only be narrowed to the family or genus, or in some cases were reported as an unknown agent (e.g., ‘unknown nematode’). The bacterial pathogen *Dermatophilus congolensis* had caused dermatitis in the highest number of wildlife species (*n* = 18) and across the greatest number of wildlife orders (Table 3); followed by the *Parapoxvirus* genus (*n* = 13), and the mite species *Demodex* and *Notoedres*, both causing dermatitis in eight species of wildlife (Table 3). All other causal agents caused dermatitis in four or less species (Table S4).

## 5. Causes of Dermatitis across Wildlife Orders

**Figure 4:**
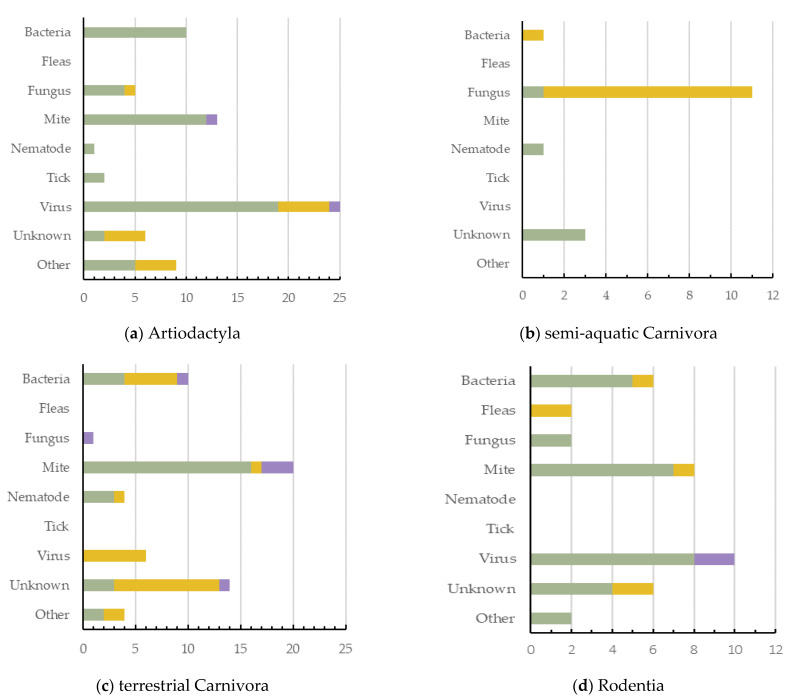

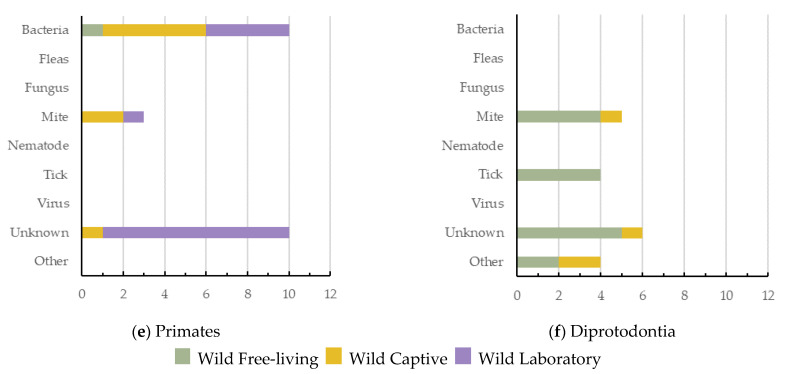
The causes of dermatitis diagnosed for each wildlife order, and for each captivity status (wild free-living, wild captive, and wild laboratory), plotted against the number of definitive dermatitis diagnosis/cases: (**a**,**c**) 0–25 cases; (**b**,**d**–**f**) 0–15 cases.

Artiodactyla had the highest number of dermatitis cases reported (*n* = 71, 27.6%, 5 ecozones), with viruses being the greatest cause of dermatitis in this order (Figure 4a). Semi-aquatic Carnivora had the lowest number of cases reported (*n* = 16; 6.2%; 2 ecozones), with fungus being their most common cause (Figure 4b). Terrestrial Carnivora was second highest (*n* = 59; 23.0%; 5 ecozones), with mite cases being most prevalent (Figure 4c). Rodentia had 36 dermatitis cases reported (14.0%; 3 ecozones), with viruses being the greatest cause of dermatitis in this mammal order (Figure 4d). Primates had 23 dermatitis cases reported (8.9%; 4 ecozones), and the most prominent causes reported were either bacteria or unknown (Figure 4e). Diprotodontia had 19 dermatitis cases reported (7.4%; 2 ecozones), often with an unknown cause (Figure 4f).

Primates had the highest number of species and cases of dermatitis for wild laboratory mammals (Figure 4e). Terrestrial Carnivora had the highest number (14) of unknown dermatitis causes. Interestingly, mites have been shown to cause dermatitis across all orders except semi-aquatic Carnivora. Bacteria was responsible for dermatitis cases in all orders except Diprotodontia, with viruses reported as a cause of dermatitis in only three (Figure 4).

## 6. Threatened Species Reported with Dermatitis

Of the 108 wild mammal species reported as having dermatitis, 65.7% (*n* = 71) are regarded as ‘Least Concern’ by the IUCN (2021) and another 10.2% (*n* = 11) are regarded as ‘Near threatened’. These two groups have been evaluated by the IUCN as species not under immediate threat of extinction and, therefore, we omitted these 82 species from further analysis on conservation concerns. We also omitted two species from the bat family Vespertilionidae, and one species of goral, *Naemorhedus griseus*, due to insufficient data on their IUCN conservation status (2.8%, *n* = 3). The other 21.3% (*n* = 23) of species are classified by IUCN as ‘Vulnerable’ (12%, *n* = 13), ‘Endangered’ (4.6%, *n* = 5) or ‘Critically Endangered’ (4.6%, *n* = 5) (Table 4). There were 45 cases of dermatitis reported across the 23 threatened species: nine cases of Critically Endangered (CR), seven cases of Endangered (EN), and 29 cases of Vulnerable (VU) (Table 3). There were no species that were classified as Extinct, Extinct in the Wild, or Regionally Extinct reported having dermatitis.

Terrestrial Carnivora had the highest number of reported dermatitis cases (*n* = 16), in six threatened species, followed by Artiodactyla with reported cases (*n* = 9) in four threatened species (Table 4). Dermatitis cases were reported more often in threatened species outside of their endemic ecozone and within captivity (57.8%, *n* = 26) (Table 4). There was no consistent cause of dermatitis identified for the 45 cases reported across all threatened species (Table S5). In threatened species, treatment of dermatitis was attempted in 26 cases (58%), which consisted of medical intervention for the cure of the cause of dermatitis (e.g., antibiotics; ivermectin), or for mitigation of clinical signs (e.g., removal of lesions) if the cause was chronic (e.g., Feline Herpes Virus). Treatment was successful in most instances (*n* = 17) and was not attempted in 19 cases (42%) (Table S5).

## 7. Discussion

Overall, there were more than 60 causal agents of dermatitis reported in wildlife, showing that this clinical sign can manifest from a variety of causes. However, the highest single proportion of reported cases was of an unknown cause. This may be since identifying the causal agent is recognized as a key challenge in the literature, and there are a variety of sampling techniques needed to correctly detect the dermatitis agent. Of the 60 definitive causes of dermatitis, mites were the most common across all species. Bacteria were the second highest cause of dermatitis across wild mammalian species, and this was driven by *Dermatophilus congolensis* (causing dermatophilosis) infection. There were a higher number of cases reported in free-ranging wildlife, and captivity had a greater role to play for the development of dermatitis in IUCN threatened species. However, the causes of dermatitis are rarely reported as a conservation issue for threatened species. Reporting biases are recognized in the ecozone Nearctic and the mammalian orders of terrestrial Carnivora and Artiodactyla.

### 7.1. The Main Causes of Dermatitis in Mammalian Wildlife: Mites, Bacteria and Viruses

Due to the wide taxonomic range of mites’ (such as *Sarcoptes scabiei*) host species, it is unsurprising that they account for the highest percentage of diagnosed dermatitis cases in the greatest number of wildlife species [35,36,37]. Mites were the predominant cause of dermatitis reports in five of the six mammal orders, and, across these, over 15 genera of mites were represented with the top three: *Demodex* sp., *Notoedres* sp. and *Sarcoptes scabiei*. *Demodex* mites are commensal, found in hair follicles across an array of taxa, including humans. However, under favorable environments, they can become opportunistic and an over proliferation can cause the skin condition demodicosis, which can cause dermatitis [38]. Conversely, the *Notoedres sp*. and *Sarcoptes scabiei* are both from the Sarcoptidae family, and are highly contagious pathogens that can infest a wide variety of animal orders [35,36,37]. *Notoedres sp*. can cause notoedric mange, and is associated with the development of dermatitis, particularly in rodents and felids, which was supported by the findings in our review [37]. In fact, both mites can cause an array of dermatological signs, from alopecia, inflammation, to even secondary infections and death. *S. scabiei* causes sarcoptic mange and is an emerging infectious disease for some species, exerting animal welfare and conservation pressures [35,36]. However, given the wide host range of this mite species, it was surprising this mite was not the leading causal agent in our review. This may be because dermatitis is not a representative clinical sign of mange, or clinical signs were not specifically mentioned in *S. scabiei* studies.

Given their global ubiquity and wide host range, it was unsurprising that viruses and bacteria were the second highest causative agents of dermatitis cases [39,40]. Viruses were mostly represented by the *Parapoxvirus* (genus), in Artiodactyla and Rodentia. High reporting of this cause may be since the *Parapoxvirus* is found worldwide and can be transmitted between domestic mammals and wildlife in the Artiodactyla order [41]. The most common bacteria species was *Dermatophilus congolensis* and bacteria from the genus *Staphylococcus*. Some bacteria, such as *Staphylococcus sp.,* are a part of normal skin microflora, and certain host and environmental factors may cause these commensal bacteria to become problematic and cause skin conditions, such as atopic dermatitis [42]. *Dermatophilus* *congolensis* (causing dermatophilosis) bacterium caused dermatitis across the most species; these bacteria are found globally, have a wide host range, and the epidemiology is broadly known in the literature [24,39,43]. Dermatophilosis has caused dermatitis in species as diverse as the meadow jumping mouse (*Zapus hudsonius*) [29] and ground squirrels (*Urocitellus columbianus columbianus*) [44] from the order Rodentia; owl monkey (*Aotus trivirgatus*) [45] and orangutans (*Pongo pygmaeus pygmaeus*) [26] from Primates; polar bears (*Ursus maritimus*) [46] and racoons (*Procyon lotor*) [47] from terrestrial Carnivora; and white-tail deer (*Odocoileus virginianus*) [48] from Artiodactyla, to name a few.

### 7.2. Captivity Status and Dermatitis in Wild Mammals

Dermatitis was reported to occur in wild free-living mammals more often than their wild laboratory or captive counterparts. This may be due to under reporting of dermatitis in captive wildlife, treatment regimens for wildlife in captivity, or biases towards the reporting of free-living individuals. Dermatological problems are common in captive wildlife, ranging from secondary infection due to poor husbandry to self-inflicted lesions subsequent to stereotypic behaviour [49,50]. Therefore, it is possible due to their commonality, skin problems are treated without being reported in the literature; and skin issues in wild animals are deemed more important. Furthermore, dermatological issues in wild captive animals may be treated before the signs of disease become advanced enough to warrant reporting, since individuals within captivity or laboratory settings are often quarantined and treated for common diseases as a precaution and are subject to routine examinations for signs of disease [51,52], whereas free-living individuals may be more likely to show later stages of dermatitis clinical signs since early treatment is unlikely.

Wild free-living mammals may be more likely to be reported with dermatitis or show later stages of clinical signs since: (i) there are no preventative steps in stopping spread and limited veterinary intervention; (ii) they come into contact with many more species and individuals which might have pathogen causing dermatitis (such as other mammals, domestic animals and livestock) [53]; and (iii) they have unregulated environmental factors, which vary from contact with unusual plants [54] to opportunistic pathogens [55].

Primates had the highest reported cases of dermatitis for wild laboratory, owing to their regular use as an experimental subject. For wild laboratory mammals, where almost half of the dermatitis causes were ‘unknown’, it is likely that commensal and non-pathogenic parasites were a prevailing problem, due to stress associated with an unnatural, confined environment [51]. In fact, such speculation was made for both captive and laboratory animals in Van Horn et al. [56] and Steinmetz et al. [6] where unknown dermatitis was presumed psychogenic of origin.

### 7.3. Threatened Species with Dermatitis

Generally, reported cases of dermatitis in the literature largely described the occurrence of dermatitis in the species, or in a particular part of a species’ range, for the first time. There were few reports of dermatitis being a primary concern for the conservation of a species, despite our identification of 23 threatened species reported as having dermatitis. The causes of dermatological lesions reduce fitness and thus constitute another pressure on threatened populations, with many species driven to extinction often due to compounding anthropogenic activities [57]. However, many threatened species’ articles only briefly mentioned causes of dermatitis as a conservation concern or that the mammal is endangered (e.g., [5,46,58]). Reports which focused on dermatitis causes as a conservation threat for a species, for example Munson et al. [5], Witte et al. [59] and Van Horn et al. [56], discussed dermatitis induced health problems and mortality could affect captive population sustainability and husbandry management. However, overall, there was no pattern in reporting treatment success or failure for threatened species. Pathogens causing dermatitis were also identified as a conservation threat to the red squirrel (*Sciurus vulgaris*), San Joaquin Kit Fox (*Vulpes macrotis mutica*), and Amargosa vole (*Microtus californicus scirpensis*) despite these mammals not being identified by the IUCN as at-risk species [21,23,60,61]. Therefore, it is important to consider that (1) some mammals may not have been classified as threatened when dermatitis was reported in the past, and (2) some species and subspecies may be missed by the IUCN and, as the classification is updated, more species may be added to the threatened categories in the future.

The threatened species cases in our review show that 33 dermatitis cases (total of 45) were in captivity. This may be due to the routine observations for captive individuals, compared to their free-living counterparts. However, we determine that there may be multiple compounding factors which may cause threatened species in captivity to be more likely to exhibit dermatitis, such as: lack of genetic diversity, added psychological stress in captivity [56,62], exposure to alien pathogens, and environmental exacerbations [63]. Loss of genetic diversity is common in many threatened species, potentially lowering their resistance to diseases and inflammatory conditions, making them more prone to agents which can cause dermatitis [64,65,66]. Loss of genetic diversity may be one of the reasons terrestrial Carnivora had the highest number of threatened species dermatitis cases reported, because many lack the genetic diversity of other mammals [65,67]. Low genetic diversity and small population size are also a conservation concern, particularly for the management of breeding programs, and act in synergy with disease and a species’ capacity to adapt to changing environmental conditions [64,66,68,69]. Conversely, large populations of nonthreatened individuals have a greater ability to adapt to captivity stressors [63].

Physiological responses to stress, or stress induced by environmental changes, might also manifest as dermatitis in threatened species in captivity via immune suppression [62,63,67]. For example, Munson et al. [65] describe both captive and free-ranging cheetahs having the same genetic diversity; however, captive cheetahs had worse reactions to viral infections and severe inflammatory reactions to common infections, suggesting that the local environment is important in determining health trajectories of individuals, beyond just genetic susceptibility. Additionally, since the majority (70%) of dermatitis cases for captive threatened species were located outside of their endemic range; captive stressors may be exacerbated for threatened species due to exposure to alien pathogens and sub-optimal environmental conditions [69,70,71]. It is well known that the introduction of wild non-native species to foreign geographic ranges has increased potential for disease emergence and outbreaks [71,72].

For several species, the development of dermatitis as a reaction to disease is associated with genetic and behavioral features [73,74,75]. This is particularly true of rhinoceroses, which are especially prone to skin diseases and lesions [5]. Dermatitis manifestation in rhinoceroses was not due to one specific bacterial or viral infection, but was instead associated with concurrent diseases or events, where dermal erosions or ulcers are the first clinical signs of underlying health issues [5,76]. Determining the reasons why some threatened species may be more susceptible to dermatological diseases can help inform management practices and husbandry behaviors within captivity.

### 7.4. Reporting Bias for Orders of Mammalian Wildlife and across Ecozones

Our synthesis might be impacted by a reporting bias for dermatitis in the mammalian orders of Artiodactyla and terrestrial Carnivora. These orders have similar and lower number of species, at about 220 and 268, respectively, compared to Primates (300) and Rodentia (1500). We suspect that, with a higher number of species, there is a greater chance of dermatitis occurring and being opportunistically observed in that mammalian order. Potential observation bias for reporting dermatitis in larger animals may be related to chance occurrence, since dermatitis is easier to see in larger animals [77,78] or reported due to relative-attention bias [79]. Furthermore, it is known that historical biodiversity records for charismatic species, particularly large mammals, have reporting biases [80,81]. This may also explain the high number of reported dermatitis cases in both terrestrial Carnivora and Artiodactyla, and less for Rodentia, despite the order Rodentia having about six-fold more species. Additionally, Artiodactyla species are genetically similar (or, in some cases, used as) agricultural animals [82,83], or game species [48]. Clegg et al. [84] reported dermatitis for the first time in wild elk; elk potentially contracted the bacterial species *Treponema* from livestock pastures and could now be a reservoir for domestic livestock and other wild animals. This is especially the case of the genus *Parapoxvirus*, with articles on this dermatitis causing agent focusing on domestic livestock species or human infections from wildlife [41,85]. This implies that Artiodactyla species’ proximity to humans and the potential for economic consequences might also explain why this order is the primary focus of dermatitis reports.

Biogeographic realms have been used as the broadest scale for identifying and planning the conservation of species [86,87] and biotic processes [88,89]. However, applying macroecological generalities to complex systems such as infectious disease has been viewed as an unresolved challenge and, until recently, biogeography has rarely been included in management of human or veterinary health [90,91]. For certain dermatitis causes, an ecozone’s environmental attributes could inhibit or exacerbate dermatitis severity, spread of causes and growth of lesions. Therefore, there is no doubt that biogeographical analysis offers a potentially important explanatory role that can provide insight into spatial patterns of multiple systems [90,92]. However, our synthesis on the literature to date suggest that funding and economics play a greater role in the reporting of dermatitis in ecozones. Indeed, our overview of this research suggests that opportunistic reports have led to an artificial trend of dermatitis in the Nearctic [79,93]. For example, dermatophilosis is more likely to occur in relatively low altitude areas with tropical and subtropical climates [39]. Therefore, in theory, dermatophilosis would be more likely to be reported in the Afrotropic and Neotropic ecozones [26]; however, more cases of dermatophilosis were reported within the Nearctic. Geographical bias in reporting has been found in other studies [94,95,96], supporting the potential for a skew towards reports in the Nearctic ecozone.

### 7.5. Limitations of Reviewing Clinical Signs of Diseases or Irritants

Our review provides a valuable synthesis of the use of the term dermatitis and patterns in mammalian wildlife. Indeed, such syntheses on common causes of dermatitis have been produced for domestic and livestock animals [97,98]; however, in wildlife, focus has been primarily on either (i) a certain type of agent affecting the wildlife, e.g., *parapoxvirus* or *Neospora caninum* infection in wildlife (e.g., [41,99,100]) or (ii) on pathogens affecting individual species (e.g., captive pinnipeds) which are, by chance, or perhaps due to visual dermatological conditions (e.g., [18,59,101]). Our unique synthesis, while not completely exhaustive, is representative of published information across different mammalian wildlife groups for a clinical sign (dermatitis), which has, to date, been overlooked but is important to consider.

However, we have identified several limitations in reviewing clinical signs of pathogen agents. Firstly, the variable use of clinical signs in diseases or irritants is a key challenge in undertaking syntheses. For example, histopathological descriptions of dermatitis may be used without applying the term explicitly. Due to this, the search term for dermatitis could underestimate the number of articles with dermatitis, since it does not capture cases with ‘dermatitis like’ clinical signs.

Furthermore, the term dermatitis may not be used in the title, abstract or key words because it is: (i) not a key feature of the histopathology of that pathogen; (ii) unclearly defined, such as a dermatitis like term; or (iii) the clinical signs are not mentioned at all, most likely because it is a well-known pathogen. The latter may be the case for Sarcoptic mange, caused by the mite *Sarcoptes scabiei* in over 100 mammalian species. However, we only found *S. scabiei* as the cause of dermatitis in seven wildlife species in our review, and, therefore, dermatitis is either not a term used for normally describing mange, or, because it is a well-known pathogen, the histological signs are not described in the abstract. In another example, White Nose Syndrome (WNS) in bats has been described as having histologically identifiable infiltrative fungal dermatitis [22]. However, we had no articles with WNS in our synthesis. Two articles in our review were screening for WNS in bats; however, they describe dermatitis as the main clinical signs of other pathogen infections [55,102].

Conversely, the clinical signs of disease would be more likely to be within the title and abstract if the clinical sign in question is unknown, since the dermatological origin is not identified. To combat this, we suggest that the clinical aspects of the skin diseases and irritation be described in the abstract of papers, regardless of whether the pathogen is well known. Despite limitations of reporting of clinical signs of skin diseases and irritants, our synthesis identifies the overall causes and patterns of dermatitis in wild mammalian species.

## 8. Conclusions

This review has identified that, overall, there seems to be bias towards reporting dermatitis cases when the cause is unknown or when presented in a species for the first time. For some species, it was noted that the underlying causal agents were first investigated due to the visibility of dermal lesions (e.g., [5,78]). Through the valuable compendium of reports, we have additionally identified that, when the causal agent of dermatitis is unknown, reports may be more likely to include dermatitis within the title and abstract (since the dermatological symptoms are in question and the origin not identified). Furthermore, the search term for dermatitis could also underestimate the number of articles and cases showing dermatitis, since the term might not be included in the title or abstract of articles dealing with a disease or agent for which dermatitis is usually just one of several possible manifestations. Conversely, for threatened species with dermatitis, 15 cases from 45 were of unknown causes (Table S5). This is perhaps because the identification of unknown causes of dermatitis, and the successful treatment of dermatitis lesions, regardless of cause, are of particular interest to threatened species given their parlous conservation status.

In general, dermatitis is rarely reported as a conservation issue; however, we have discovered that some threatened species may be more likely to exhibit dermatitis in captivity, and, for others, diseases often manifest, at least in part, as dermatitis. This review highlights that, in some cases, species of concern are declining due to specific reasons that are clinically revealed as dermatitis. However, dermatitis reporting in wild semi-aquatic and terrestrial mammals remains subject to many biases. As such, future case studies of diseases should: (i) document the main clinical signs and manifestations of the disease or causative agent in the abstract of reports, and (ii) encompass a range of dermatological conditions, since an animal may be suffering a form of dermatitis but was classified as another skin disorder (e.g., dermatophilosis). With our suggestions, we can bridge cross-disciplinary gaps between veterinary, genetics, captive management, and conservation, and further research can determine common spatial patterns of dermatological diseases for wild mammals.

## Figures and Tables

**Table 1:** Article Data Extraction: for each article, we collected information on the manuscript, the individuals per species, and the dermatitis exhibited by each species.

Manuscript	Mammal (Per Species)	Dermatitis (Per Species)
Title	Number of individuals	Type
Author(s)	Captivity status	Location of dermatitis
Year	Country	Clinical signs
Journal	Conservation status	Definitive Cause
Key terms		

**Table 2:** Overall proportion of causes of dermatitis in terrestrial and semi-aquatic mammalian wildlife. This shows the cause of dermatitis, the diagnosis proportion (% out of 257 cases), the number of unique species per cause, and the number of ecozones for which the cause of dermatitis was reported in. For further analysis, causes with ‘*’ were combined into ‘Other’ category.

Cause	Percentage Definitive Diagnosis (*n*)	Species per Cause	Ecozone
Apicomplexan *	1.2% (3)	2	1
Bacteria	16.3% (42)	29	6
Bacteria & Fungus *	0.8% (2)	1	1
Diptera *	1.2% (3)	3	2
Ectoparasite (unknown) *	0.4% (1)	1	1
Fleas	0.8% (2)	2	1
Fungus	9.0% (23)	15	3
Louse *	0.8% (2)	2	2
Mineral deficiency *	0.4% (1)	1	1
Mite	21.4% (55)	34	5
Mite & Fungus *	0.4% (1)	1	1
Mite & Nematode *	0.4% (1)	1	1
Nematode	2.7% (7)	6	2
Plant *	1.6% (4)	4	2
Protozoa *	0.4% (1)	1	1
Tick	2.3% (6)	6	2
Unknown	23.7% (61)	35	5
Virus	16.3% (42)	15	4

* Other.

**Table 3:** The top seven causal agents of dermatitis in wildlife species, with all other causes effecting four or less species (Table S4). For each causal agent, the etiological category, the number of species the agent was reported to cause dermatitis in, and the number of orders is shown.

Causal Agent	Category	Species	Orders
*Dermatophilus congolensis*	Bacteria	18	6
*Parapoxvirus* (genus)	Virus	13	2
*Demodex* sp. (genus)	Mite	8	4
*Notoedres* sp. (genus)	Mite	8	3
*Sarcoptes scabiei*	Mite	7	2
*Staphylococcus* sp. (genus)	Bacteria	7	6
*Malassezia sp.* (genus)	Fungus	6	2

**Table 4:** International Union for Conservation of Nature’s (IUCN) Red List of threatened wild mammal species reported as having dermatitis. The 23 species categorized as ‘Critically Endangered’ (CR), ‘Endangered’ (EN), and ‘Vulnerable’ (VU) are shown, including their order and total number of cases. The number of dermatitis cases within endemic ecozones are shown for each species and the number of cases within non-endemic ecozones. Both ecozone categories are separated into the following captivity statuses of: Wild Captive (WC), Wild Free-living (WFL) and Wild Laboratory (WL). * WFL has one reported case within a non-endemic ecozone (*), and WL has no reported cases across non-endemic ecozones, so the columns have been removed to simplify the table.

IUCN (2021) Threatened Species	Threatened Category	Order	Total Cases	Endemic Ecozone Cases			Non-Endemic Ecozone Cases *
				WFL	WC	WL	WC
*Canis rufus* (red wolf)	CR	terrestrial Carnivora	1		1		
*Dasyprocta mexicana* (Mexican agouti)	CR	Rodentia	1		1		
*Diceros bicornis* (black rhinoceros)	CR	Perissodactyla	5	2			3
*Mustela lutreola* (European Mink)	CR	terrestrial Carnivora	1				1
*Pongo pygmaeus pygmaeus* (Northwest Bornean orangutan)	CR	Primates	1				1
*Ailurus fulgens* (red panda)	EN	terrestrial Carnivora	2				2
*Elephas maximus* (Asian Elephant)	EN	Other (Elephantidae)	2		1		1
*Pentalagus furnessi* (Amami rabbit)	EN	Lagomorpha	1	1			
*Petrogale persephone* (Proserpine rock wallaby)	EN	Diprotodontia	1	1			
*Symphalangus syndactylus* (siamang)	EN	Primates	1				1
*Acinonyx jubatus* (cheetah)	VU	terrestrial Carnivora	5		1		4
*Alouatta palliata* (Mantled Howler Monkey)	VU	Primates	1	1			
*Budorcas taxicolor tibetana* (Sichuan takin)	VU	Artiodactyla	1				1
*Hippopotamus amphibius* (Nile hippopotamus)	VU	Artiodactyla	3				3
*Lagothrix lagotricha* (woolly monkey)	VU	Primates	1				1
*Macaca fascicularis* (crab-eating macaque)	VU	Primates	2				2 *
*Petrogale penicillata* (brush-tailed rock-wallaby)	VU	Diprotodontia	1	1			
*Phascolarctos cinereus* (koala)	VU	Diprotodontia	1	1			
*Rangifer tarandus* (caribou)	VU	Artiodactyla	4	1	2	1	
*Rhinoceros unicornis* (Indian rhinoceros)	VU	Perissodactyla	2				2
*Rusa unicolor* (Sambar)	VU	Artiodactyla	1				1
*Tremarctos ornatus* (Andean bear)	VU	terrestrial Carnivora	3				3
*Ursus maritimus* (polar bear)	VU	terrestrial Carnivora	4	2	1		1
		Total	45	10	7	1	26(*1)

## Data Availability

The data presented in this study are available in the Supplementary Materials section.

## Supplementary Materials

The following are available online at https://www.mdpi.com/article/10.3390/ani11061691/s1, Search terms S1: The search terms used to identify the relevant literature in each database, Table S2: Complete dataset of all 216 articles that reported wildlife dermatitis, Table S3: All individually reported dermatitis cases for each order not presented in the synthesis, Table S4: Etiological agents responsible for the causes of dermatitis across mammalian wildlife species, Table S5: Each reported threatened species dermatitis cases and their corresponding IUCN (2021) threatened categories, concurrent disease, dermatitis treatment, and whether the treatment resolved dermatitis.
