# Supplementary material for: The Patterns and Causes of Dermatitis in Terrestrial and Semi-Aquatic Mammalian Wildlife
Source: Animals (Basel). 2021 Jun 6;11(6):1691. doi: 10.3390/ani11061691 (PMC8228222; doi:10.3390/ani11061691)
Supplement: Supplementary file 1 [file animals-11-01691-s001.zip › animals-1243578-supplementary.pdf]

SUPPLEMENTARY MATERIAL

Search terms S1. The search terms used to identify the relevant literature in each database.

Web of Science: advanced, all years, all databases selected:
TS= ("dermatitis" OR "exudative dermatitis") AND TS= "wildlife"
Result of 162 (19th Dec 2019)
Result of 269 (12th March 2021)

Scopus: advanced
TITLE-ABS-KEY ("dermatitis" OR "exudative dermatitis" AND "wildlife")
Result of 98 (19th Dec 2019)
Result of 86 (12th March 2021)

Google Scholar
"dermatitis" OR "exudative dermatitis" AND "wildlife"
Result of 18,700 and retain first 100 (19th Dec 2019)
Result of 19,300 and retain first 100 (12th March 2021)

Table S2. Complete dataset of all 216 articles that reported wildlife dermatitis. For each article we collected information on the manuscript, the individuals per species, and the dermatitis exhibited by each species, of which, there were 257 cases in total.

Paper Title	Author(s)	Year	Journal Title	Species	Country/Region (location of species)	Animal Origin	No. Animals	Conservation for the species/other species mentioned?	Dermatitis Type	Clinical signs/Dermatitis	Location of Dermatitis	Cause: Difinitive Diagnoses	Cause: Specilative/Hypothses	Key terms
Acral lick dermatitis in a jackal (*Canis aureus*)	Yeruham, Israel<br>Nyska, Abraham	1998	Journal of zoo and wildlife medicine: official publication of the American Association of Zoo Veterinarians	*Canis aureus* (jackal)	Israel	Wild Captive	1	no	Acral lick dermatitis	Lesion	NA	NA	Psychogenic	NA
Alopecia in Rhesus macaques correlates with immunophenotypic alterations in dermal inflammatory infiltrates consistent with hypersensitivity etiology	Kramer, J.<br>Fahey, M.<br>Santos, R.<br>Carville, A.<br>Wachtman, L.<br>Mansfield, K.	2010	Journal of Medical Primatology	*Macaca mulatta* (Rhesus macaques)	United States of America	Wild Laboratory	36	no	Chronic hypersensitivity dermatitis; Atopic dermatitis	Alopecia	Localized to forearms and lower legs, occasionally spread to shoulders and/or thighs, some on torso	NA	Psychogenic	allergy, dermatitis, hair-loss, macaque skin
The Alopecia-Syndrome of Spectacled Bears (Tremarctos ornatus)–What Do We Know, What Can We Do?	Jäger, K<br>Langguth, S<br>Einspanier, A<br>Schachtner, M<br>Bechstein, N<br>Schoon, H-A	2013	Journal of Comparative Pathology	*Tremarctos ornatus* (Andean bear)	Europe	Wild Captive	96	yes	Dermatitis	generalized alopecia with severe pruritus, eborrhoea sicca and lichenification, perivascular der-matitis	NA	NA	allergic skin disorder, genetic or social issues	NA
Alternaria fungal dermatitis in free-ranging javelina (*Pecari tajacu*)	Shender, Lisa A.<br>Gerhold, Rick<br>Sanchez, Susan<br>Keel, M. Kevin	2011	Journal of Wildlife Diseases	*Pecari tajacu* (javelina)	United States of America	Wild Free-Living	1	no	Dermatitis	Fungal dermatitis and cellulitis. A pus-filled tract within the rostrum. Hyperkeratotic. Proliferative fibrous connective tissue	rostrum, snout, eyes, feet, capari	Fungus - (*Alternaria sp*.)	NA	NA
The Andean bear alopecia syndrome may be caused by social housing	Van Horn, R. C.<br>Sutherland-Smith, M.<br>Bracho Sarcos, A. E.<br>Thomas, G.<br>Shanks, J. A.<br>Owen, M. A.	2019	Zoo biology	*Tremarctos ornatus* (Andean bear)	United States of America	Wild Captive	20	yes	Hypersensitivity dermatitis	Pruritus, alopecia, mucopurulent ocular discharge or conjunctivitis and changes to the epidermis include thickening, roughness, and increased pigmentation	Face and periocular region as well as the caudal lumbar region and the limbs	NA	Psychogenic	alopecia, captivity, epidemiology, ex situ, *Tremarctos ornatus*
Anticoagulant exposure and notoedric mange in bobcats and mountain lions in urban southern California	Riley, Seth P. D.<br>Bromley, Cassity<br>Poppenga, Robert H.<br>Uzal, Francisco A.<br>Whited, Lynn<br>Sauvajot, Raymond M.	2007	Journal of Wildlife Management	*Lynx rufus* (bobcat), *Puma concolor* (mountain lion)	United States of America	Wild Free-living	21 - (19 bobcat, 2 mountain lion)	yes	Dermatitis (mange)	proliferative dermatitis with hyperkeratosis, epidermal scaling	head, shoulders, body, hind legs	Mite - (*Notoedres cati*)	NA	anticoagulant rodenticides, bobcat, fragmentation, mountain lion, multiple stressors, notoedric mange, southern California, synergistic effects, toxicology, urbanization.
Arcanobacterium phocae infection in mink (*Neovison vison*), seals (*Phoca vitulina, Halichoerus grypus*) and otters (*Lutra lutra)*	Nonnemann, Bettina<br>Chriel, Mariann<br>Larsen, Gitte<br>Hansen, Mette Sif<br>Holm, Elisabeth<br>Pedersen, Karl	2017	Acta veterinaria scandinavica	*Neovison vison* (mink)	Denmark	Wild Captive	14	no	pododermatitis; dermatitis	Acute profound suppurative and necrotizing pododermatitis. Subacute suppurative and necrotizing dermatitis with dry crusts around nares and hyperkeratosis of the surrounding epidermis. Severe superficial suppurative and necrotizing pododermatitis. Chronic profound suppurative and necrotizing dermatitis.	feet, head, lumbar	Bacteria - (*Arcanobacterium phocae*)	NA	*Arcanobacterium phocae, Arcanobacterium phocisimile,* Mink, Seal, Otter
Assessing causes and significance of red squirrel *(Sciurus vulgaris*) mortality during regional population restoration: An applied conservation perspective	Shuttleworth, Craig M<br>Lisa Signorile, A<br>Everest, David J<br>Paul, Duff<br>Lurze, Peter Ww	2015	Hystrix	*Sciurus vulgaris* (red squirrels)	Wales	Wilde Free-Living	2	yes	Exudative dermatitis	Squirrelpox-like lesions	Lower lip, side of lips, digits	NA	Bacteria - (*Staphylococcus aureus*)	Red squirrel, *Sciurus vulgaris,* mortality, adenovirus, Wales,
Association of a lukM-positive clone of *Staphylococcus aureus* with fatal exudative dermatitis in red squirrels (*Sciurus vulgaris*)	Simpson, V. R.<br>Davison, N. J.<br>Kearns, A. M.<br>Pichon, B.<br>Hudson, L. O.<br>Koylass, M.<br>Blackett, T.<br>Butler, H.<br>Rasigade, J. P.<br>Whatmore, A. M.	2013	Veterinary Microbiology	*Sciurus vulgaris* (red squirrels)	England, Bailiwick of Jersey	Wild Free-Living	10 (4 IOW, 6 Jersey)	no	Exudative dermatitis	Exudative scabby lesions, inflamation and sloughing of the skin of the feet and ischemic necrosis of digits. exudative, ulcerative, necrotic dermatitis with epidermal hyperplasia and hyperkeratosis and numerous colonies of gram-positive cocci, both in the exudate and within intradermal pustules	Mouth and/or nose, and some cases eyelids. feet and digits	NA	Bacteria - (*Staphylococcus aureus*)	*Sciurus vulgaris*, Dermatitis, *Staphylococcus aureus* CC49, LukM gene
Atypical dermatophytosis in 12 North American porcupines (*Erethizon dorsatum*) from the Northeastern United States 2010-2017	Needle, David B.<br>Gibson, Robert<br>Hollingshead, Nicholas A.<br>Sidor, Inga F.<br>Marra, Nicholas J.<br>Rothenheber, Derek<br>Thachil, Anil J.<br>Stanhope, Bryce J.<br>Stevens, Brian A.<br>Ellis, Julie C.<br>Spanswick, Shelley<br>Murray, Maureen<br>Goodman, Laura B.	2019	Pathogens	*Erethizon dorsatum* (North American porcupines)	United States of America	Wild Free-living	12	no	dermatitis, dermatophytosis	Lesions consisted of diffuse severe epidermal hyperkeratosis and mild hyperplasia with mild lymphoplasmacytic dermatitis and no folliculitis	dorsum, face, tail, limbs	Fungus - (*Trichophyton sp./Arthroderma benhamiae)*	NA	dermatophyte, porcupine, Erethizon, fungus, metagenomics, fungal genetics, molecular diagnostics
Berlin squirrelpox virus, a new poxvirus in red squirrels, Berlin, Germany	Wibbelt, Gudrun<br>Tausch, Simon H.<br>Dabrowski, Piotr W.<br>Kershaw, Olivia<br>Nitsche, Andreas<br>Schrick, Livia	2017	Emerging infectious diseases	*Sciurus vulgaris* (red squirrels)	Germany	Wild Free-living	10	yes	exudative and erosive-to-ulcerative dermatitis	moist crusty skin lesions.	nose, digits, tail, genital regions	Virus - (Poxvirus family)	NA	squirrelpox, poxvirus, squirrel, Sciuridae, Berlin, Germany, skin disease, dermatitis, viruses, red squirrels, Sciurus vulgaris, Berlin squirrelpox virus
Candidiasis in captive pinnipeds	Dunn, J. L.<br>Buck, J. D.<br>Spotte, S.	1984	Journal of the American Veterinary Medical Association	*Halichoerus grypus* (gray seals), *Phoca vitulina* (harbor seals), *Mirounga angustirostris* (northern elephant seal)	United States of America	Wild Captive	16 [7 - (gray seals), 8 - (harbor seals), , 1 - (northern elephant seal)]	yes	Dermatitis	purulent nasal discharge, inflammation of the lips at the mucocutaneous junction, periocular alopecia, vaginitis and dermatitis.	skin leasions around the eyes, flippers, and abdomen	Fungus - (*Candida albicans*)	NA	NA
A case of exfoliative dermatitis in a captive southern white rhinoceros (*Ceratotherium simum simum*)	Bauwens, Luc<br>De Vroey, Charles<br>De Meurichy, Walter	1996	Journal of Zoo and Wildlife Medicine	*Ceratotherium simum simum* (southern white rhinoceros)	Belgium	Wild Captive	1	no	Exfoliative dermatitis	Skin lesions/abrasions enlarging to irregular dermal erosions up to 20cm in length , detaching edges and hypermic surface covered with brown/red exudate	Back	Fungus - (*Malassezia pachydermatis* and *Candida parapsilosis* yeasts)	NA	Southern white rhinoceros, *Ceratotherium simum simum*, exfoliative dermatitis, *Malassezia pachydermatis*, *Candida parapsilosis*, natamycin
A case of parapoxvirus infection in wild Japanese serows (*Capricornis crispus*) in Ishikawa prefecture	Yata, S	1996	Japanese Society of Zoo and Wildlife Medicine	*Capricornis crispus* (Japanese serow)	Japan	Wild Free-Living	1	no	Contagious pustular dermatitis	Abscesses, keratinization on the skin, reddish knots	Left fore and hind limbs, lips	Virus - (parapoxvirus)	NA	Contagious pustular dermatitis, Japanese serow (*Capricornis crispus*), Parapox virus
A case of suspected contact dermatitis in a juvenile cynomolgus monkey (*Macaca fascicularis*)	Morris, Joanne<br>Etheridge, Marcia	2008	Journal of medical primatology	*Macaca fascicularis* (cynomolgus monkey)	United States of America	Wild Captive	1	no	Eosinophilic dermatitis	Ruptured blisters, crusts, and papules on the extremities and face	Extremities and face, oral lesions	NA	NA (contact dermatitis susepected)	eosinophilia, juvenile cynomolgus macaque, vesicular dermatitis
Causes of red squirrel (*Sciurus vulgaris*) mortality in England	Duff, JP<br>Haley, P<br>Wood, R<br>Higgins, RJ	2010	Veterinary Record	*Sciurus vulgaris* (red squirrels)	England	Wild Free-living	6	yes	dermatitis	dermatitis	ventral body and head	Louse - (*Neohaematopinus sciuri*)	NA	NA
Characterization of dermatologic changes in geriatric rhesus macaques	Huneke, R. B.<br>Foltz, C. J.<br>Vandewoude, S.<br>Mandrell, T. D.<br>Garman, R. H.	1996	Journal of Medical Primatology	*Macaca mulatta* (Rhesus macaques)	United States of America	Wild Laboratory	13	no	Subacute dermatitis/ ulcerative dermatitis/ pervascular dermatitis/ acanthotic dermatitis	Dermal lesions, erythematous skin, wrinkling, focal skin scaling, thinning of hair, foot calluses, and exudative lesions	Thigh, neck, chest, foot	NA	Age	skin-aging-endocrinedermatitis, dermatology
Chorioptic mange in a wild Japanese serow	Shibata, A.<br>Yachimori, S.<br>Morita, T.<br>Kanda, E.<br>Ike, K.<br>Imai, S.	2003	Journal of Wildlife Diseases	*Capricornis crispus* (Japanese serow)	Japan	Wild Free-living	1	yes	dermatitis	Parakeratotic hyperkeratosis lesions, mange, alopecia, marked lichenification, accumulation of crust, and fissurin	legs, thorax, ventral abdomen and perineum, axilla and inguinal regions	Mite - (*Chorioptes bovis*)	NA	*Capricornis crispus, Chorioptes bovis*, chorioptic mange, Japanese serow, mites.
Chromobacteriosis in a Chinese red panda (*Ailurus fulgens styani*)	Dyer, Nw<br>Krogh, Df<br>Devold, R<br>Wilson, Sl<br>White, Dg	2000	Journal of veterinary diagnostic investigation	*Ailurus fulgens styani* (Chinese red panda)	United States of America	Wild Captive	1	no	Necrotising dermatitis	Skin and subcutaneous tissue from around the interscapular abscess showed necrotizing dermatitis and cellulitis.	Skin	Bacteria - (*Chromobacterium violaceum*)	NA	bacteria, Chromobacterium, case studies, Florida, North Dakota
Chromobacterium violaceum Infection in a Free-ranging Howler Monkey in Costa Rica	Baldi, M.<br>Morales, J. A.<br>Hernandez, G.<br>Jimenez, M.<br>Alfaro, A.<br>Barquero-Calvo, E.	2010	Journal of Wildlife Diseases	*Alouatta palliata* (Howler monkey)	Costa rica	Wild Free-Living	1	no	Ulcerative dermatitis	Skin lesions, skin ulcers	Extremities	Bacteria - (*Chromobacteria violaceum*)	NA	Bacterial infection, *Chromobacterium violaceum*, opportunistic pathogen, wild primate

Chronic dermatitis in nutria in Louisiana	Chabreck, Robert H<br>Thompson, Roger B<br>Ensminger, Allan B	1977	Journal of wildlife diseases	*Myocastor coypus* (nutria)	United States of America	Wild Free-Living	5	no	Chronic dermatitis	Lesions, necrosis of the epidermis, thickened skin, inflamation and becterial and fungal infections	Ventral neck and chest, and upper portion of the front legs	Plant matter - (*Bidens laevis*)	NA	NA
Chronic eosinophilic dermatitis associated with persistent feline herpes virus infection in cheetahs (*Acinonyx jubatus*)	Munson, L<br>Wack, R<br>Duncan, M<br>Montali, Rj<br>Boon, D<br>Stalis, I<br>Crawshaw, Gj<br>Cameron, Kn<br>Mortenson, J<br>Citino, S	2004	Veterinary pathology	*Acinonyx jubatus* (cheetah)	United States of America	Wild Captive	20	yes	Ulcerative and eosinophilic dermatitis	Cutaneous lesions, erythematous, ulcerated plaques. a white, eosinophil-rich exudate was present in many cases	Lesions also occurred commonly at the commissures of the lips, top of the head, distal forelegs, flank, tail, and footpads	Virus - (feline herpes virus 1 (FHV1))	NA	*Acinonyx jubatus*, cheetahs, de rmatitis, eosinophils, f eline herpes virus 1, pseudoepitheliomat ous hyperplasia
Chronic pruritic dermatitis in asthmatic monkeys: A subhuman primate analogue of atopic dermatitis?	Patterson, Roy<br>Harris, Kathleen	1981	International Archives of Allergy and Immunology	*Macaca mulatta* (Rhesus macaques)	United States of America	Wild Laboratory	2	no	Atopic dermatitis	Chronic, generalized pruritus and dermatitis.Skin lesions, thickened, scaling, lichenified dermatitis	Flexural surfaces of the forearms	NA	Physiological - asthmatic monkeys	NA
Clinical and pathological characterization of northern elephant seal skin disease	Beckmen, K. B.<br>Lowenstine, L. J.<br>Newman, J.<br>Hill, J.<br>Hanni, K.<br>Gerber, J.	1997	Journal of Wildlife Diseases	*Mirounga angustirostris* (northern elephant seal)	United States of America	Wild Free-living	207	no	Ulcerative dermatitis	skin lesions were characterized by patchy to extensive alopecia and hyperpigmentation, punctate or coalescing epidermal ulceration, and occasionally, massive skin necrosis. Microscopic lesions included ulcerative dermatitis with hyperkeratosis, squamous metaplasia and atrophy of sebaceous glands.	body surface	NA	exposure to polyhalogenated aromatic hydrocarbons (PHAHs) which indude PCBs and polyhrominated bipheny!s (PBBs).	Northern elephant seal, Mirounga angustirostris, hyperkeratosis, squamous metaplasia, ulcerative dermatitis, skin disease, PCB, DDT.
Clinical mange of the black bear, *Ursus americanus*, associated with *Ursicoptes americanus* (Acari: *Audycoptidae*)	Yunker, Ce<br>Binninger, Ce<br>Keirans, Je<br>Beecham, J<br>Schlegel, M	1980	Journal of Wildlife Diseases	*Ursus americanus* (American black bear)	United States of America	Wild Free-Living	1	no	Dermatitis	Alopecia, chronic lesions,heavy exfoliation, large scales and apparent pruritis	Head, muzzle, neck, thorax, front legs	Mite - (*Ursicoptes americanus*)	NA	NA
Clinical Picture and Antibody Response to Experimental Sarcoptes scabiei var. vulpes Infection in Red Foxes (*Vulpes vulpes*)	Bornstein, S.<br>Zakrisson, G.<br>Thebo, P.	1995	Acta Veterinaria Scandinavica	Vulpes vulpes (red fox)	Sweden	Wild Laboratory	3	no	Dermatitis	alopecia, crusts, excoriations, hyperkeratosis, dermatitis, lesions, excematous dermatitis	back, thorax, hock, hind leg, shoulder, tail, head	Mite - (*Sarcoptes scabiei*)	NA	sarcoptic mange; red fox; serodiagnosis; ELISA.
Comparison of a cheetah herpesvirus isolate to feline herpesvirus type 1	Scherba, G<br>Hajjar, AM<br>Pernikoff, DS<br>Sundberg, JP<br>Basgall, EJ<br>Leon-Monzon, M<br>Nerurkar, L<br>Reichmann, ME	1988	Archives of virology	Acinonyx jubatus (cheetah)	United States of America	Wild Captive	3	yes	severe ulcerative dermatitis	Chronic, multifocal, ulcerative cutaneous lesions	NA	Virus - (Cheetah herpesviruses ChHV)	NA	NA
Contagious ecthyma in an adult Dall sheep (*Ovis dalli dalli*) in Alaska	Smith, Tc<br>Heimer, We<br>Foreyt, Wj	1982	Journal of Wildlife Diseases	*Ovis dalli dalli* (Dall sheep)	United States of America	Wild Free-Living	1	no	Dermatitis	Numerous sessile, slightly crusted lesions, raised mats	Thigh, mammary glands and teats	Virus - (Contagious ecthyma (CE), Orf virus)	NA	NA
Contagious ecthyma in bighorn sheep and mountain goat in western Canada	Samuel, Wm<br>Chalmers, Ga<br>Stelfox, Jg<br>Loewen, A<br>Thomsen, Jj	1975	Journal of Wildlife Diseases	*Ovis canadensis canadensis* (bighorn sheep), *Oreamnos americanus* (mountain goat)	Canada	Wild Free-Living	3 (2 sheep,1 goat)	no	Pustular dermatitis	Severe lesions numerous irregular, confluent, raised, crusted, dark brown to black lesions located along the margins of the lips.	Muzzel and lips	Virus - (Contagious ecthyma (CE), Orf virus)	NA	NA
Contagious ecthyma in wild thar in the south island	Kater, Joan C<br>Hansen, Nf	1962	New Zealand Veterinary Journal	*Hemitragus jemlahicus* (thar)	New Zealand	Wild Free-Living	1	no	Pustular dermatitis	Extensive scabby lesions, moist proliferative dermatitis between the digits and extending to the heels	Lips, nostrils, and feet	Virus - (Contagious ecthyma (CE), Orf virus)	NA	NA
Contagious ecthyma, Rangiferine Brucellosis, and lungworm infection in a Muskox (*Ovibos moschatus*) from the Canadian Arctic, 2014	Tomaselli, M.<br>Dalton, C.<br>Duignan, P. J.<br>Kutz, S.<br>Van Der Meer, F.<br>Kafle, P.<br>Surujballi, O.<br>Turcotte, C.<br>Checkley, S.	2016	Journal of Wildlife Diseases	*Ovibos moschatus* (muskox)	Canada	Wild Free-Living	1	no	Proliferative dermatitis,	Proliferative dermatitis, contagious ecthyma, or orf (parapoxvirus)	Muzzel and fetlocks	Virus - (Parapoxvirus)	NA	Brucella suis biovar 4; Parapoxvirus; Protostrongylidae; food safety; food security; public health; wildlife health and disease surveillance; zoonoses
Contagious mucocutaneous dermatitis of the mountain hare (*Lepus timidus*): pathology and cause	Saari, Seppo Am<br>Rudbäck, Eeva<br>Niskanen, Mirjami<br>Syrjälä, Paula<br>Nylund, Minna<br>Anttila, Marjukka	2005	Journal of wildlife diseases	*Lepus timidus* (mountain hares)	Finland	Wild Free-Living	53	no	Contagious mucoctuaneous dermatitis	Lesions, necrosis, ulceration and inflamation.	Proliferative, crusting lesions of the mucocutaneous junctions of the mouth and nose	Virus - (poxvirus)	NA	Mountain hare, mucocutaneous dermatitis, poxvirus, Staphylococcus warnerii, ulcerative dermatitis
Corynebacterium freneyi bacterial septicemia secondary to contagious ecthyma in a wild muskox (*Ovibos moschatus*)	Rothenburger, J. L.<br>Di Francesco, J.<br>Leclerc, L. M.<br>van der Meer, F.<br>Tomaselli, M.<br>Zabek, E.<br>Kutz, S. J.	2021	Journal of Wildlife Diseases	*Ovibos moschatus* (Wild Muskox)	Canada	Wild Free-living	1	yes	Ulcerative and proliferative dermatitis	Contagious ecthyma (CE), Orf virus: Necrotizing and proliferative suppurative dermatitis with folliculitis and abscesses	haired skin, lips, and mucocutaneous junction of the muzzle	Virus - (Parapoxvirus)	NA	NA
Cutaneous Dermatophilosis in a Meadow Jumping Mouse (*Zapus hudsonius*)	Caron, T. J.<br>Artim, S. C.<br>Israelsen, W. J.<br>Holcombe, H. R.<br>Fox, J. G.<br>Bakthavatchalu, V.	2018	Comparative Medicine	*Zapus hudsonius* (meadow jumping mouse)	United States of America	Wild Captive	1	no	Exudative and proliferative dermatitis	Multilaminated crusts covering the epidermis, extensive scaling of the face, limbs, and tail and severe edema of the paws. Digital hematomas and white scales, scabs, and crusts affecting the majority of nonhaired skin	Face, limbs, tail, paws, skin	Bacteria - (*Dermatophilus congolensis*)	NA	NA
Cutaneous streptothricosis (dermatophiliasis) in owl monkeys	King, Nwj<br>Fraser, Ceo<br>Garcia, Fg<br>Wolf, La<br>Williamson, Martha E	1971	Laboratory Animal Science	*Aotus trivirgatus* (owl monkey)	United States of America	Wild Laboratory	5	no	Proliferative and exudative dermatitis	Lesions resembling papillomas, raised, alopecia, wart like masses, brown thick crusts, exudate	Face (eyelids, chin, lips), extremities, hind limbs, tail	Bacteria - (*Dermatophilus congolensis*)	NA	NA
Cutaneous streptothricosis (dermatophilosis) in a woolly monkey	Fraser, Ceo<br>Garcia, Fg	1971	Primate Zoonosis Surveillance	*Lagothrix lagotricha* (woolly monkey)	United States of America	Wild Captive	1	no	Dermatitis	Wart-like skin lesions, dark brown crust, proliferative	Skin	Bacteria - (*Dermatophilus congolensis*)	NA	NA
Cyclic dermatitis associated with *Fusarium sp* infection in pinnipeds	Montali, Richard J<br>Bush, R Mitchell<br>Strandberg, Jd<br>Janssen, Dl<br>Boness, Daryl J<br>Whitla, Joan C	1981	Journal of the American Veterinary Medical Association	*Zalophus californianus* (Californian sea lion), *Halichoerus grypus* (gray seal)	United States of America	Wild Captive	6 (3 sea lions, 3 seals)	no	Dermatitis	Lesions were papular or nodular	Facem trunk, flippers	Fungus - (*Fusarium sp*)	NA	NA
Demodectic mange in a white-tailed deer from Walker County, Texas	Turner, Jc<br>Cano, J	2014	Journal of medical entomology	*Odocoileus virginianus* (white-tailed deer)	United States of America	Wild Free-Living	1	no	Exudative dermatitis	Alopecia, dry erythmatous escharotic inflammation, thickened epidermis and dermis, lesions, epilation	Rump, posterior flanks, withers, neck, chest	Mite - (*Demodex sp*)	NA	NA
Demodectic mange, dermatophilosis, and other parasitic and bacterial dermatologic diseases in free-ranging white-tailed deer (*Odocoileus virginianus*) in the United States from 1975 to 2012	Nemeth, N. M.<br>Ruder, M. G.<br>Gerhold, R. W.<br>Brown, J. D.<br>Munk, B. A.<br>Oesterle, P. T.<br>Kubiski, S. V.<br>Keel, M. K.	2014	Veterinary Pathology	*Odocoileus virginianus* (white-tailed deer)	United States of America	Wild Free-Living	88 (37 Demodectic mange; 19 Dermatophilosis; 13 dermatitis most commonly with bacteria; all others had different forms of ectoparasites causing dermatitis).	no	Nodular dermatitis, exudative dermatitis, ulcerative dermatitis, ectoparasite dermatitis, eosinophilic dermatitis	Cases of demodectic mange were defined as those in which animals had gross lesions suggestive of demodicosis (ie, variable alopecia, thickened skin, crusting, scaling, lichenification, or seborrhea). Dermatophilosis identified with gross lesions usually consisted of alopecia with crusting and erythema on the face and ears as well as the distal limbs	50% of the body, head, neck, limbs, shoulders, flanks, dorsum, ventrum, axillae. Face, ears, limbs.	Mite - (*Demodex sp*.); Bacteria - (*Dermatophilus congolensis*, *Arcanobacterium haemolyticum*, *Staphylococcus* and *Streptococcus spp*); Mites - (*Chorioptes texanus*); Louse - (*Tricholipeurus spp*); Ticks (*Amblyomma maculatum* and *Amblyomma americanum*)	NA	Demodex spp, dermatopathology, Dermatophilus congolensis, Odocoileus virginianus, white-tailed deer
Demodicosis in black bears (*Ursus americanus*) from Florida	Forrester, Donald J<br>Spalding, Marilyn G<br>Wooding, John B	1993	Journal of Wildlife Diseases	*Ursus americanus* (American black bear)	United States of America	Wild Free-Living	5	no	Dermatitis	Severe alopecia, hyperkeratosis, follicular pustules	Body, face, chest, trunk and limbs	Mite - (*Demodex sp*)	NA	Demodicosis, Demodex sp., black bear, Ursus americanus, Florida, case report.
Demodicosis in chamois (*Rupicapra rupicapra* subsp. *Rupicapra*) in the Italian Alps, 2013–14	Salvadori, C.<br>Formenti, N.<br>Trogu, T.<br>Lanfranchi, P.<br>Papini, R. A.<br>Poli, A.	2016	Journal of Wildlife Diseases	*Rupicapra rupicapra* (chamois)	Italy	Wild Free-living	5	no	Dermatitis	moderate crusts, intramural folliculitis,	head, trunk	Mite - (*Demodex sp*)	NA	NA
Dermatitis and rhinosinuitis caused by Curvularia species in a chinese goral (*Naemorhedus griseus*)	Ter Beest, Julia M<br>Garner, Michael M<br>Love, David M<br>Bradway, Daniel S<br>Daniels, Joshua B<br>Surman, Sean T<br>Neiffer, Donald L<br>Ramer, Jan C	2020	Journal of Zoo and Wildlife Medicine	*Naemorhedus griseus* (Chinese goral)	United States of America	Wild Captive	1	yes	Dermatitis	cutaneous, lesions, pyogranulomatous dermatitis	pinnae	Fungus - (*Curvularia* sp.)	NA	Chinese goral, *Curvularia s p.*, dematiaceous, fungal, *Naemorhedus griseus*, phaeohyphomycosis.
Dermatitis associated with infestation of a trombiculid mite, *Leptotrombidium miyajimai*, in an Amami Rabbit (*Pentalagus furnessi*)	Kubo, Masahito<br>Sato, Hiroshi<br>Hattori, Shosaku<br>Kuraishi, Takeshi	2014	Journal of wildlife diseases	*Pentalagus furnessi* (Amami rabbit)	Japan	Wild Free-Living	1	yes	Dermatitis	Dermatitis with alopecia and excoriation. Epidermis moderately thickened with hyperkeratosis	Ventral abdomen, axillary, and inguinal regions	Mite (*Leptotrombidium miyajimai*)	NA	NA
Dermatitis caused by dermatophilus congolensis in a Zoo Polar Bear (*Ursus maritimus*)	Eo, K. Y.<br>Kwon, O. D.	2014	Pakistan Veterinary Journal	*Ursus maritimus* (polar Bear)	South Korea	Wild Captive	1	no	Dermatitis	Severe exudative dermatitis with thick scab formation, matted hair, thickening of the skin, crust formation	Face and whole body	Bacteria - (*Dermatophilus congolensis*)	NA	Dermatitis Dermatophilus Dermatophilus congolensis Polar bear

Dermatitis caused by Dermatophilus congolensis in polar bears (*Thalarctos maritimus*)	Smith, Cf	1973	Veterinary Record	*Thalarctos maritimus* (Polar bear)*[*Ursus maritimus*]	New Zealand	Wild Captive	2	no	Dermatitis	Skin debris	Skin	Bacteria - (*Dermatophilus congolensis*)	NA	NA
Dermatitis caused by Filaria taxideae in a lesser panda	Gardiner, Ch<br>Loomis, Mr<br>Britt, Jr Jo<br>Montali, Rj	1983	Journal of the American Veterinary Medical Association	*Ailurus fulgens* (lesser panda)	United States of America	Wild Captive	1	no	Dermatitis	Alopecia, hyperkeratotic, skin lesions with pus-filled subcutaneous abscesses	Back, left shoulder, thorax, scrotum	Nematode - (*Filaria taxideae*)	NA	NA
Dermatitis caused by group G beta-hemolytic Streptococcus in Nile hippos (*Hippopotamus amphibius*)	Clyde, VL<br>Wallace, RS<br>Pocknell, AM	1998	American Association Of Zoo Veterinarians	*Hippopotamus amphibius* (Nile hippopotamus)	United States of America	Wild Captive	3	no	Dermatitis	cracking, wounds, lesions, suppurative, multifocal epidermitis, severe hyperkeratosis and multifocal parakeratosis	nose, flanks, shoulders, face limbs, trunk, mandible	NA	The dermatitis in these cases is likely the result of opportunistic infection of skin lesions by normal skin flora.	NA
Dermatitis caused by Malassezia pachydermatis in a California sea lion (*Zalophus californianus*)	Chermette, R	1998	The Veterinary Record	*Zalophus californianus* (Californian sea lion)	France	Wild Captive	1	no	Focal Dermatitis	Cutaneous lesions, wheals, orthokeratotic hyperkeratosis of the epidermis	Flanks and chest	Fungus - (*Malassezia pachydermatis*)	NA	NA
Dermatitis in free-living common brushtail possums (*Trichosurus vulpecula*)	Hemsley, S<br>Canfield, Pj	1994	Australian Veterinary Practioner	*Trichosurus vulpecula* (common brushtail possum)	Australia	Wild Free-Living, Wild Captive	6 (1 wildlcaptive)	no	Dermatitis	Exuditve, alopecia, scab formation, ulceration, puritic lesions, thickening of the epidermins	Hip, tail, lumbar, thighs, head, forelimbs, flanks, ears, lips	NA	NA	NA
Dermatitis in seals caused by *D. congolensis*	Frese, K<br>Weber, A	1971	Berliner und Münchener Tierärztliche Wochenschrift	*Otaria byronia* (South American sea lion)	Germany	Wild Captive	2	no	Dermatitis	Eczematous skin, lesions, ruffled hair	head, neck, limbs	Bacteria - (*Dermatophilus congolensis*)	NA	
Dermatitis in the black rhinoceros (*Diceros bicornis*) due to filariasis	Schulz, KCA<br>Kluge, EB	1960	Journal of the South African Veterinary Association	*Diceros bicornis* (Black Rhinoceros)	South Africa	Wild Free-living	1	no	Dermatitis	lesions, ulcerative wounds, exfoliation, erosions, pustule and crust formation, ulceration and haemorrhage	shoulders,	Nematode - (unknown)	NA	NA
Dermatologic investigation of alopecia in rhesus macaques (*Macaca mulatta*)	Steinmetz, Hanspeter W<br>Kaumanns, Werner<br>Dix, Ilona<br>Neimeier, Karl-Albrecht<br>Kaup, Franz-Josef	2005	Journal of Zoo and Wildlife Medicine	*Macaca mulatta* (Rhesus macaques)	Germany	Wild Laboratory	183	no	Dermatits	Mild epidermal hyperkeratosis and mild perivascular<br>dermatitis	Back and extremities were most commonly affected	NA	NA	Rhesus monkey, Macaca mulatta, hair loss, alopecia
Dermatopathy in captive hippopotamus (*Hippopotamus amphibious*)	Helmick, Ke<br>Rush, Em<br>Ogburn, Al<br>Trupkiewicz, Jg<br>Garner, M	2007	Proc. Am. Assoc. Zoo Vet. Pg	*Hippopotamus amphibius* (Nile hippopotamus)	United States of America	Wild Captive	3	no	Ulcerative dermatitis	Crackling, peeling, or vesicle lesions. ulcers which drain blood, serum or purulent material	Neck, limbs, thorax, and abdomen, dorsum, toes, and perineal region.	NA	Bacteria and Fungus	NA
Dermatophilosis (Cutaneous streptothricosis) in Kafue lechwe (*Kobus leche kafuensis*)	Pandey, G. S.<br>Mweene, A.<br>Suzuki, A. K.<br>Nambota, A.<br>Kaji, T.	1994	Journal of Wildlife Diseases	*Kobus leche kafuensis* (Kafue lechwe)	Zambia	Wild Free-Living	2	no	Exudative dermatitis	Thickening of the skin, crusts, and nodfule formation	Most of the body	Bacteria - (*Dermatophilus congolensis*)	NA	Dermatophilus congolensis, Kafue lechwe, Kobus leche kafuensis, Zambia
Dermatophilosis (cutaneous streptothricosis) in owl monkeys	Fox, Jg<br>Campbell, Lh<br>Reed, C<br>Snyder, Sb<br>Soave, Oa	1973	Journal of the American Veterinary Medical Association	*Aotus triviragtus* (owl monkey)	United States of America	Wild Captive	5	no	Ulcerative dermatitis	Lesions, crusts, ulceration, alopecia, papillomatous whitish brown lesions, alopecia, exudate, debris	Head, eyelids, ear, mouth	Bacteria - (*Dermatophilus congolensis*)	NA	NA
Dermatophilosis among wild raccoons in New York State	Salkin, If<br>Gordon, Ma<br>Stone, Wb	1976	Journal of the American Veterinary Medical Association	*Procyon lotor* (Raccoons)	United States of America	Wild Free-Living	8	no	Dermatits	Encursted lesions	Eyes, snout, tarsal and carpal regions	Bacteria - (*Dermatophilus congolensis*)	NA	NA
Dermatophilosis in a mule deer, *Odocoileus hemionus* (*Rafinesque*), from Wyoming	Williams, Elizabeth S<br>Pier, Ac<br>Wilson, Roger W	1984	Journal of wildlife diseases	*Odocoileus hemionus* (mule deer)	United States of America	Wild Free-Living	3	no	Exudative dermatitis	Scabs and exudative crusts, ulcerative skin	Skin of ears, chin, axilla	Bacteria - (*Dermatophilus congolensis*)	NA	NA
Dermatophilosis in owl monkeys	Mcclure, Hm<br>Kaplan, W<br>Bonner, Wb<br>Keeling, Me	1971	Sabouraudia: Journal of Medical and Veterinary Mycology	*Aotus trivirgatus* (owl monkey), *Maraca mulatta* (Rhesus monkey), *Macaca fascicularis* (cynomolgus), *Saimiri sciureus* (squirrel monkeys)	United States of America	Wild Captive, Wild Laboratory	8 (2 of each species, wild captive are the 2 owl monkeys)	no	Exudative dermatitis	Exudative lesions, crusts, scales, alopecia, thick brown crusts	Legs, tail, back, ears, arms, head	Bacteria - (*Dermatophilus congolensis*)	NA	NA
Dermatophilosis in two polar bears	Newman, Ms<br>Cook, Rw<br>Appelhof, Wk<br>Kitchen, H	1975	Journal of the American Veterinary Medical Association	*Thalarctos maritimus* (Polar bear)*[*Ursus maritimus*]	United States of America	Wild Captive	2	no	Dermatitis	Yellowing of hair, greasiness, pruritus, encurstation of skin, lesions, scab, exudate and necrotic debris	Skin, trunk, face	Bacteria - (*Dermatophilus congolensis*)	NA	NA
Dermatophilosis of alpine chamois (*Rupicapra rupicapra*) in Italy	De Meneghi, Daniele<br>Ferroglio, Ezio<br>Bollo, Enrico<br>Leon Vizcaino, L<br>Moresco, A<br>Rossi, Luca	2002	Schweizer Archiv für Tierheilkunde	Rupicapra rupicapra (chamois)	Italy	Wild Free-living	2	no	Proliferative dermatitis	crusted lesions, scab, hair loss, scaliness and scabs,	leg, nose,	Bacteria - (*Dermatophilus congolensis*)	NA	Dermatophilus congolensis, chamois (Rupicapra rupicapra), strawberry footrot, dermatophilosis, Italian Alps
Dermatophilus congolensis infections in wildlife in New York State	Salkin, If<br>Stone, Wb<br>Gordon, Ma	1981	Journal of clinical microbiology	*Mephitis mephitis* (striped skunk), *Marmota monax* (woodchuck), *Procyon lotor* (raccoon)	United States of America	Wild Free-Living	3 (1 of each sp)	no	Exudative dermatitis	Crusts, scales, alopecia, lesions	Skunk - axillae, wrists, ankles, digits woodchuck - head, neck, legs, feet, abdomen, back raccoon - eyes, snout, neck, hocks, sides, legs	Bacteria - (*Dermatophilus congolensis*)	NA	NA
Dermatophilus dermatitis enzootic in deer in New York State and vicinity	Gordon, Ma<br>Salkin, If<br>Stone, Wb	1977	Journal of wildlife diseases	*Odocoileus virginianus* (white-tailed deer)	United States of America	Wild Free-Living	15	no	Dermatitis	Portions of skin bearing crusts or scabs, eroded or denuded areas of skin, pustules and detached scabs.	Ears	Bacteria - (*Dermatophilus congolensis*)	NA	NA
Dermatophilus infection in Columbian Ground Squirrels (*Citellus columbianus columbianus*)	Wobeser, G<br>Gordon, Ma	1969	Bulletin of the Wildlife Disease Association	*Urocitellus columbianus columbianus* (Columbian ground squirrels)	Canada	Wild Free-Living	2	no	Dermatitis	Skin lesions, thick crusts, keratinized skin, scabs, alopecia	Nose, cheeks, hind legs, perineal region	Bacteria - (*Dermatophilus congolensis*)	NA	NA
Dermatophytosis caused by Trichophyton mentagrophytes in the Southern Chamois (*Rupicapra pyrenaica*) in the Eastern Pyrenees	Marco, I.<br>Lopez-Olvera, J. R.<br>Gibert, P.<br>Abarca, L.<br>Gauthier, D.<br>Lavín, S.	2007	Zoonoses and Public Health	*Rupicapra pyrenaica* (Pyrenean chamois)	France, Spain	Wild Free-living	2	no	Dermatitis	alopecia, scalling, crusting skin lesions	Dorsum and hind limbs, horns, eyes, nose, tail, sternum	Fungus - (*Trichophyton mentagrophytes*)	NA	NA
Detection of Dichelobacter nodosus in wild ungulates (*Capra ibex ibex* and *Ovis aries musimon*) and domestic sheep suffering from foot rot using a two-step polymerase chain reaction	Belloy, Luc<br>Giacometti, Marco<br>Boujon, Patrick<br>Waldvogel, Andreas	2007	Journal of Wildlife Diseases	*Capra ibex ibex* (Alpine ibex)	Switzerland	Wild Free-living	25	no	Exudative dermatitis	lesions, pododermatitis, exudative dermatitis, multifocal sole ulcers, foot rot	feet	Bacteria - (*Dichelobacter nodosus*)	NA	Capra ibex ibex, Dichelobacter nodosus, foot rot, mouflon, Ovis aries musimon,<br>PCR, wild ungulates
Detection of mecC-Positive Staphylococcus aureus (CC130-MRSA-XI) in Diseased European Hedgehogs (*Erinaceus europaeus*) in Sweden	Monecke, S.<br>Gavier-Widen, D.<br>Mattsson, R.<br>Rangstrup-Christensen, L.<br>Lazaris, A.<br>Coleman, D. C.<br>Shore, A. C.<br>Ehricht, R.	2013	Plos One	*Erinaceus europaeus* (European hedgehog)	Sweden	Wild Free-Living	1	no	Diffuse dermatitis	Thickening of the skin and prominent crusts affecting mostly skin areas free of spine. Skin lesions included ulcerations and formation of serocellular crusts	Areas free of spines	Bacteria - (*Staphylococcus aureus*)	NA	Hedgehogs, Staphylococcus aureus, Methicillin-resistant Staphylococcus aureus, Staphylococcus, Polymerase chain reaction, Wildlife, Microarrays, Neutrophils
Development of a case definition for clinical feline herpesvirus infection in cheetahs (*Acinonyx jubatus*) housed in zoos	Witte, Carmel L<br>Lamberski, Nadine<br>Rideout, Bruce A<br>Fields, Victoria<br>Teare, Cyd Shields<br>Barrie, Michael<br>Haefele, Holly<br>Junge, Randall<br>Murray, Suzan<br>Hungerford, Laura L	2013	Journal of Zoo and Wildlife Medicine	*Acinonyx jubatus* (cheetah)	United States of America	Wild Captive	21	yes	dermatitis	skin lesions, dermatitis	lesions on the face, legs, tail, and inside the<br>mouth	Virus - feline herpesvirus (FHV)	NA	Acinonyx jubatus, case definition, cheetah, epidemiology, feline herpesvirus, upper respiratory<br>disease
Diagnosis and treatment of Neospora caninum–associated dermatitis in a red fox (*Vulpes vulpes*) with concurrent Toxoplasma gondii infection	Dubey, Jitender P<br>Whitesell, Leah E<br>Culp, William E<br>Daye, Sharon	2014	Journal of Zoo and Wildlife Medicine	*Vulpes vulpes* (red fox)	United States of America	Wild Captive	1	no	Neosporosis dermatitis	Generalized crusty plaques resembling mange, skin lesions with many intralesional protoxoal tachyzoites. neosporosis, granulomatous dermatitis, hyperkeratosis with neutrophilic crusting.	Body, face, chest, trunk and limbs	Protozoa - (*Neospora caninum*)	NA	NA

Diseases diagnosed in red foxes from the southeastern United States	Little, Susan E<br>Davidson, William R<br>Howerth, Elizabeth W<br>Rakich, Pauline M<br>Nettles, Victor F	1998	Journal of wildlife diseases	*Vulpes vulpes* (red fox)	United States of America	Wild Free-living	33	no	Dermatitis	severe dermatitis, gross lesion was diffuse, marked hyperkeratosis and alopecia with severe dermal lichenification. Hyperkeratotic dermatitis	NA	Mite - (*Sarcoptes scabiei)*	NA	Disease, mortality factors, red fox, sarcoptic mange, survey, vulpes vulpes
Diseases in free-ranging bats from Germany	Mühldorfer, Kristin<br>Speck, Stephanie<br>Wibbelt, Gudrun	2011	BMC Veterinary Research	Vespertilionidae (bat family)	Germany	Wild Free-living	7	yes	Dermatitis	moderate purulent dermatitis, focal ulcerative necrotizing dermatitis	skin	NA	2 mixed bacterial, and/or bacterial-fungus; 5 had unknown origin	NA
Dual infection of a white-tailed deer by *Dermatophilus congolensis* and *Alternaria alternata*	Salkin, If<br>Gordon, Ma<br>Stone, Wb	1975	Journal of the American Veterinary Medical Association	*Odocoileus virginianus* (white-tailed deer)	United States of America	Wild Free-Living	1	no	Exudative dermatitis	Encrusted lesions, scaling and alopecia	Hocks, flanks, back, ears, eyes, torso	Bacteria - (*Dermatophilus congolensis*), & Fungus - (*Alternaria alternata*)	NA	NA
Ear mange mites (*Notoedres muris*) in black and Norway rats (*Rattus rattus and Rattus norvegicus*) from inner-city Vancouver, Canada	Anholt, H.<br>Himsworth, C.<br>Rothenburger, J.<br>Proctor, H.<br>Patrick, D. M.	2014	Journal of Wildlife Diseases	*Rattus rattus* (black rat), *Rattus norvegicus* (Norway rat)	Canada	Wild Free-living	15 (13 *Rattus rattus*, 2 *Rattus norvegicus*)	no	Proliferative dermatitis	exhibited epidermal hyperplasia and hyperkeratosis with intracorneal pustules and mild lymphoplasmacytic dermatitis	ears, face, legs, tail	Mite - (*Notoedres muris*)	NA	Ecology, ear mange, *Notoedres muris*, Rattus, urban.
Ectoparasitic dermatitis in free-ranging swamp wallabies (*Wallabia bicolor*) in New South Wales	Portas, T. J.<br>Crowley, A.<br>Hufschmid, J.	2009	Australian Veterinary Journal	*Wallabia bicolor* (swamp wallaby)	Australia	Wild Captive	3	no	Ectoparasitic dermatitis	Extensive dermatological lesions, alopecia and hyperkeratosis of the pinnae and tail, patchy alopecia over the thorax and abdomen and large symmetrical areas of alopecia and hyperkeratosis adjacent to the tail base. additionally each animal had localised crusting, alopecia and papules	Pinnae and tail, patchy alopecia over the thorax and abdomen and large symmetrical areas of alopecia and hyperkeratosis adjacent to the tail base, thighs and the inguinal region	Louse - (*Heterodoxus ualabat* i)	NA	dermatology; Diabolicoptes; Heterodoxus ualabati; lice; mites; swamp wallaby
Effects of Habitat, Host Sex and Age on the Parasites of *Trichosurus Caninus* (Marsupialia: *Phalangeridae*) in North-Eastern New South Wales	Presidente, Pja<br>Barnett, Jl<br>How, Ra<br>Humphreys, Wf	1982	Australian journal of zoology	*Trichosurus caninus* (Short-eared possum), *Trichosurus vulpecula* (brush-tailed possum)	Australia	Wild Free-Living	56 *T. caninus*<br>3 *T. vulpecula*	no	Focal Dermatitis	Lesions, oedema, scar or scab formation at tick attachment sites	Scrotum, pouch, mandible, ears	Tick - (*Ixodes spp.*)	NA	NA
Efficacy of in-feed formulation ivermectin against psoroptes sp in bighorn sheep	Foreyt, W. J.	1993	Journal of Wildlife Diseases	*Ovis canadensis canadensis* (bighorn sheep)	United States of America	Wild Laboratory	2	no	Exudative dermatitis	Psoroptic mange, alopecia, and exudative dermatitis with scabs	Ears, face and neck.	Mite (*Psoroptes sp*)	NA	Psoroptes sp., psoroptic mange, scabies, drug trial, ivermectin, Rocky Mountain bighorn sheep, Ovis canadensis canadensis, experimental infestation.
The emergence of squirrelpox in Ireland	Mcinnes, Cj<br>Coulter, L<br>Dagleish, Mp<br>Deane, D<br>Gilray, J<br>Percival, A<br>Willoughby, K<br>Scantlebury, M<br>Marks, N<br>Graham, D	2013	Animal Conservation	*Sciurus vulgaris* (red squirrels)	Northern Ireland	Wild Free-Living	2	yes	Exudative dermatitis	Gross lesions, severe dermatitis, scab, alopecia, ulceration of the epidermis	Eyelids, footpads, chin	Virus - (squirrelpox)	NA	squirrelpox; SQPV; grey squirrels; red squirrels; Ireland.
Epidemiological and postmortem findings in 262 red squirrels (*Sciurus vulgaris*) in Scotland, 2005 to 2009	Larose, Jp<br>Meredith, Al<br>Everest, Dj<br>Fiegna, C<br>Mcinnes, Cj<br>Shaw, Dj<br>Milne, Em	2010	Veterinary Record	*Sciurus vulgaris* (red squirrels)	Scotland	Wild Free-Living	33	yes	Exudative dermatitis	Ulceration, scab formation	Feet, face, ventrum	Virus - (squirrelpox)	NA	NA
Epidemiology of clinical feline herpesvirus infection in zoo-housed cheetahs (*Acinonyx jubatus*)	Witte, C. L.<br>Lamberski, N.<br>Rideout, B. A.<br>Vaida, F.<br>Citino, S. B.<br>Barrie, M. T.<br>Haefele, H. J.<br>Junge, R. E.<br>Murray, S.<br>Hungeford, L. L.	2017	Journal of the American Veterinary Medical Association	*Acinonyx jubatus* (cheetah)	United States of America	Wild Captive	50	yes	Dermatitis	Crusted or scabbed skin ulcers, dermatitis, or skin lesions	Limited to areas of ocular and salivary secretion and where cheetahs commonly lick themselves [eg, forelimbs]	Virus - (Clinical feline herpesvirus (FHV))	NA	NA
An epizootic of besnoitiosis in captive caribou (*rangifer tarandus caribou*), reindeer (*rangifer tarandus tarandus*) and mule deer (*odocoileus hemionus hemionus*)	Glover, G. J.<br>Swendrowski, M.<br>Cawthorn, R. J.	1990	Journal of Wildlife Diseases	*Rangifer tarandus caribou* (caribou); *Odocoileus hemionus hemionus* (mule deer)	Canada	Wild Captive	31 [23 - *Rangifer tarandus caribou* (caribou); 8 - *Odocoileus hemionus hemionus* (mule deer)]	no	Dermatitis	mild thickening with slight superficial scaling to marked thickening associated with hyperpigmentation, fissuring and epidermal ulceration with serous exudation and crust formation. Severe generalized exudative dermatitis. Skin lesions, cysts	distal extremities, eyelids, skin and lips, face, legs	Apicomplexan - (*Besnoitia sp*.)	NA	Besnoitia sp., besnoitiosis, caribou, reindeer, mule deer, Rangifer tarandus caribou, Ran gifer tarandus tarandus, Odocoileus hemionus hemlonus, epidemic, insect vector
Eradication of a tropical rat mite (*Ornithonyssus bacoti*) infestation from a captive colony of endangered amargosa voles (*Microtus californicus scirpensis*)	Mantovani, S.<br>Allan, N.<br>Pesapane, R.<br>Brignolo, L.<br>Foley, J.	2018	Journal of Zoo and Wildlife Medicine	*Microtus californicus scirpensis* (Amargosa vole)	United States of America	Wild Captive	29	yes	Ulcerative pleocellular dermatitis	Round, gross, skin leasions 0.5 to 1 cm in diameter, with raised, reddened edges and central ulceration. focal erosive and mild, diffuse ulcerative pleocellular dermatitis	Cervical and scapular regions	Mite - (*Ornithonyssus bacoti*)	NA	Amargosa vole; Microtus californicus scirpensis; Ornithonyssus bacoti; captive wildlife; selamectin; tropical rat mite
Erythema multiforme in a spotted hyena (*Crocuta crocuta*)	Hanley, Christopher S<br>Simmons, Heather A<br>Wallace, Roberta S<br>Clyde, Victoria L	2005	Journal of Zoo and Wildlife Medicine	*Crocuta crocuta* (Spotted hyena)	United States of America	Wild Captive	1	no	Dermatitis	acute, severe erythematous and ulcerative dermatitis	hindquarters, feet, face, and ears	NA	either the vaccine or amoxicillin the potenital cause of erythema multiforme	Erythema multiforme, spotted hyena, Crocuta crocuta, drug reaction, canine distemper vaccine, canary pox vaccine.
Exfoliative dermatitis in the Indian rhinoceros (*Rhinoceros unicornis*), with description of a new species: Pityrosporum pachydermatis	Weidman, FD	1925	Zoological Society of Philadelphia, PA	*Rhinoceros unicornis* (Indian rhinoceros); *Diceros bicornis* (Black Rhinoceros)	United States of America	Wild Captive	3 (2. *R. unicornis*; 1 *D. bicornis*)	no	Exfoliative dermatitis	flaking skin, superfically fissured, scaling, rusty brown skin, pitting, dermatitis exfoliativa, genmeralized, marked exfoliation of scales, dry, inflammation, ulcers, pustular	hide of back, flanks, body, legs, ears, head	*R. unicornis* : Fungus - (Potentially - *Pityrosporum pachydermatis*)	*D. bicornis*: unknown	NA
Experimental deerpox infection in black-tailed deer (*Odocoileus hemionus Columbianus*)	Bildfell, R. J.<br>Thompson, K. A.<br>Moerdyk-Schauwecker, M.<br>Jin, L.<br>Wolff, P. L.<br>Gillin, C. M.	2010	Journal of Wildlife Diseases	*Odocoileus hemionus columbianus* (black-tailed deer)	United States of America	Wild Captive	7	no	Dermatitis	multifocal poxviral cutaneous lesions of erythema, papules, pustules, ulceration, and crusting	ears, neck, mouth	Virus - (*Cervidpoxvirus*)	NA	NA
Experimental infection of red foxes (*Vulpes vulpes*) with Sarcoptes scabiei var. vulpes	Mörner, Torsten<br>Christensson, Dan	1984	Veterinary Parasitology	*Vulpes vulpes* (red fox)	Sweden	Wild Laboratory	1	no	Dermatitis	alopecia, moderate dermatitis	back, tail, hindquarter skin	Mite - (*Sarcoptes scabie* i)	NA	NA
Experimental parapoxvirus infection (contagious ecthyma) in semi-domesticated reindeer (*Rangifer tarandus tarandus*)	Tryland, M.<br>Klein, J.<br>Berger, T.<br>Josefsen, T. D.<br>Das Neves, C. G.<br>Oksanen, A.<br>Asbakk, K.	2013	Veterinary Microbiology	*Rangifer tarandus tarandus* (mountain reindeer)	Norway	Wild Laboratory	14	no	Contagious pustular dermatitis	Contagious ecthyma, small white lesions proliferative dermatitis with epidermal hyperplasia, hyperkeratosis, intra-epithelial pustules and ulcer. multiple proliferative and coalescing lesions in the oral mucosa.	Mouth	Virus - (p*arapoxvirus*)	NA	Contagious ecthyma, Experimental infection, Orf, Parapoxvirus, Poxvirus, Reindeer, Wildlife
Fatal anemia and dermatitis in captive agoutis (*Dasyprocta mexicana*) infested with *Echidnophaga* fleas	Cucchi-Stefanoni, Karina<br>Juan-Sallés, Carles<br>Parás, Alberto<br>Garner, Michael M	2008	Veterinary Parasitology	*Dasyprocta mexicana* (agoutis)	Mexico	Wild Captive	3	no	Hyperplastic perivascular dermatitis	Cutaneous thickening, lesions, alopecia, puritis, ulcers	Dorsal, flank, eyelid	Fleas - (*Echidnophaga sp*.)	NA	agouti, anemia, cardiomegaly, hepatocellular necrosis, dermatitis, Echidnophaga, flea,, hypersensitivity, rodent
Filarial dermatitis in a striped skunk	Saito, Emi K<br>Little, Susan E	1997	Journal of Wildlife Diseases	*Mephitis mephitis* (striped skunk)	United States of America	Wild Free-Living	1	no	Diffuse dermatitis	Alopecia, thickening of the skin, multiple cutaneous abscesses	Dorsal aspect of head, neck and trunk.	Nematodes (*Filaria taxideae*)	NA	dermatitis, Filaria taxideae, mephitis mephitis, striped skunk
Filaria taxideae in striped skunks (*Mephitis mephitis*) of Colorado, USA, and commonly associated filarial dermatitis	Fox, K. A.<br>Martin, L. E. R.<br>Baeten, L. A.<br>Webb, B. T.<br>LeVan, I. K.<br>Ballweber, L. R.<br>Wolfe, L. L.	2013	Journal of Wildlife Diseases	*Mephitis mephitis* (striped skunk)	United States of America	Wild Free-living	4	no	Dermatitis	hyperkeratosis, skin thickening, inflammation, hemorrhage, crusting, ulceration, lesions	skin	Nematodes (*Filaria taxideae*)	NA	NA
First cases of squirrelpox in red squirrels (*Sciurus vulgaris*) in Scotland	Mcinnes, Cj<br>Coulter, L<br>Dagleish, Mp<br>Fiegna, C<br>Gilray, J<br>Willoughby, K<br>Cole, M<br>Milne, E<br>Meredith, A<br>Everest, Dj	2009	Veterinary Record	*Sciurus vulgaris* (red squirrels)	Scotland	Wild Free-Living	4	yes	Exudative dermatitis	Skin lesions, scabs, ulcers	Head, feet, prepuce	Virus - (squirrelpox)	NA	NA

First report of acariasis by Caparinia tripilis in African hedgehogs, (*Atelerix albiventris*), in Costa Rica	Moreira-Soto, Andres<br>Troyo, Adriana<br>Calderón-Arguedas, Olger	2013	Revista brasileira de parasitologia veterinaria	*Atelerix albiventris* (African hedghog)	Costa rica	Wild Captive	2	no	Pruritic dermatitis (mange)	chronic pruritic dermatitis, scabs, nearly complete loss of spines, alopecia	periorbital region, lateral surfaces of the head, the flanks, hind and fore limbs, abdominal area, and rump.	Mite - (*Caparinia tripilis*)	NA	Erinaceidae, ectoparasites, mange, Psoroptidae, Central America, African hedgehog.
The first report of massive infestation with *Lipoptena Cervi* (Diptera: *Hippoboscida*e) in Roe Deer *(Capreolus Capreolus)* in Iasi county, N-E of Romania	Lazar, M.<br>Iacob, O. C.<br>Solcan, C.<br>Pasca, S. A.<br>Lazar, R.<br>Boisteanu, P. C.	2017	Arquivo Brasileiro De Medicina Veterinaria E Zootecnia	*Capreolus capreolus* (roe deer)	Romania	Wild Free-living	4	no	Dermatitis	dermatitis, alopecia, hemorrhages	face, head, neck, lateral body parts, abdominal regions, inguinal, perianal, body	Diptera - (L*ipoptena cervi)*	NA	roe deer, Lipoptena cervi, SEM , histopathology, Iasi county
First report of straelensiosis in red foxes (*Vulpes vulpes*)	Sargo, Roberto F. J.<br>Mota, Stephanie M.<br>Loureiro, Filipa R. F.<br>Sousa, Luis M. C.<br>Silva, Filipe C.<br>Prada, Justina	2020	Veterinary Dermatology	*Vulpes vulpe*s (red fox)	Portugal	Wild Free-living	3	no	Nodular dermatitis	nonpuritic cutaneous papules	head, neck, limbs, trunk	Mite - *(Straelensia cynotis*)	NA	NA
Foot health and prevalence of Dichelobacter nodosus in 11 ungulate species at Berne Animal Park	Hoby, S.<br>Steiner, A.<br>Kuhnert, P.<br>Jost, R. Furtado<br>Guthruf, S.<br>Schoenbaechler, K.<br>Alsaaod, M.	2020	Schweizer Archiv Fur Tierheilkunde	*Bison bonasus* (European bison)	Switzerland	Wild Captive	9	no	Dermatitis	unknown agent, bison. Mange in alpaca	fore and hind limbs	NA,<br>Mite - (*Chorioptes* sp)	NA	Captive wild and domestic ungulates, Dichelobacter nodosus, Foot health, PCR, Lameness
Foot Lesions in farmed mink (*Neovison vison*):pathologic and epidemiologic characteristics on 4 Danish farms	Jespersen, A.<br>Hammer, A. S.<br>Jensen, H. E.<br>Bonde-Jensen, N.<br>Lassus, M. M.<br>Agger, J. F.<br>Larsen, P. F.	2016	Veterinary Pathology	*Neovison vison* (Mink)	Denmark	Wild Captive	520	no	Pododermatitis	lesions, hair loss, crusting, hyperkeratosis, ulcerative lesions, pyogranulomatous dermatitis, Furunculosis and granulomatous dermatitis	feet	NA	Potential hair follicle infection	callus, dermatitis, foot, hyperkeratosis, mink, plantar, skin, trichogranuloma
Fungal dermatitis in captive pinnipeds	Pollock, Christal G<br>Rohrbach, Barton<br>Ramsay, Edward C	2000	Journal of Zoo and Wildlife Medicine	*halichoerus grypus* (gray seals), *Phoca vitulina* (harbor seals), *Zalophus californianus* (California sea lion)	United States of America	Wild Captive	8 (2 gray seals, 4 harbor seals, 2 sea lions)	no	Fungal dermatitis	Erythematous, thickened, alopecic skin lesions	Face and on the flippers, particularly around the nail bed	Fungus (*Trichophyton mentagrophytes*, *Malassezia spp*., and *Yarrowia (Candida) lipolytica*)	NA	Gray seal, Phoca vitulina, harbor seal, Halichoerus grypus, sea lion, Zalophus californianus, fungal, dermatitis.
Generalized demodecosis in three sibling, juvenile rock hyraxes (*Procavia capensis*)	Sturgeon, Ginger<br>Suedmeyer, William<br>Mertins, James<br>Garner, Michael	2010	Journal of Zoo and Wildlife Medicine	*Procavia capensis* (rock hyraxes)	United States of America	Wild Captive	3	no	dermatitis	severe generalized dermatitis, nonpruritic, nonalopecic, mildly encrusted, focally ulcerated pustular nodules	dorsum, limbs, and dorsal cranium	Mite - (Demodex sp.)	NA	Demodex, doramectin, mite, Procavia capensis, rock hyrax
Genetic characterization of orf viruses isolated from various ruminant species of a zoo	Guo, J<br>Rasmussen, J<br>Wünschmann, Arno<br>De La Concha-Bermejillo, A	2004	Veterinary microbiology	*Ovibos moschatus* (muskox), *Budorcas taxicolor tibetana* (Sichuan takin)	United States of America	Wild Captive	8 Muskox, 2 Sichuan takin	no	Proliferative dermatitis	Skin lesions<br>muskox - severe, persisent, multifocal, proliferative, cauliflower like<br>sichuan takin - mild, transient, fcoal , wart like, ulcers	Skin, muzzels and lip, eyes, nose	Virus - (parapoxvirus)	NA	Orf virus (ORFV), Bovine papular stomatitisvirus (BPSV), Pesudocowpox virus (PCPV), Musk ox, sichuan takin, shetland sheep
Genetic epidemiology and pathology of raccoon-derived Sarcoptes mites from urban areas of Germany	Rentería-Solís, Z.<br>Min, A. M.<br>Alasaad, S.<br>Müller, K.<br>Michler, F.-U.<br>Schmäschke, R.<br>Wittstatt, U.<br>Rossi, L.<br>Wibbelt, G.	2014	Medical and Veterinary Entomology	*Procyon lotor* (Raccoons)	Germany	Wild Free-living	1	no	Proliferative dermatitis	severe proliferative dermatitis and alopecia	gluteal regions, thighs, knees	Mite - (*Sarcoptes scabiei*)	NA	Sarcoptes scabiei, microsatellites, pathology, raccoon, wildlife
Haematological, serum biochemical and serological features of platypuses with and without mycotic granulomatous dermatitis	Connolly, Jh<br>Obendorf, Dl<br>Whittington, Rj	1999	Australian Veterinary Journal	*Ornithorhynchus anatinus* (platypus)	Australia	Wild Free-Living	13	no	Granulomatous dermatitis	Ulcerative skin lesions	Tail, body	Fungus - (*Mucor amphibiorum*)	NA	Platypus, Mucor amphibiorum, dermatitis, haematology, serum biochemistry, ELISA
Hair-loss epizootic in moose (*Alces alces*) associated with massive deer ked (*Lipoptena cervi*) infestation	Geraghty, Dominic P.<br>Griffiths, Joshua<br>Stewart, Niall<br>Robertson, Iain K.<br>Gust, Nick	2011	Journal of Wildlife Diseases	*Alces alces* (Moose)	Norway	Wild Free-living	1	no	Dermatitis	alopecia, acute to chronic, multifocal to coalescing, eosinophilic to lymphocytic dermatitis	cheeks, neck, thorax abdomen	Diperta - *(Lipoptena cervi)*	NA	*Alces alces*, climate, deer ked, dermatitis, hair loss, *Lipoptena cervi*, population density, pathology.
Hair-loss syndrome in black-tailed deer of the Pacific Northwest	Bildfell, Robert J<br>Mertins, James W<br>Mortenson, Jack A<br>Cottam, Doug F	2004	Journal of Wildlife Diseases	*Odocoileus hemionus columbianus* (black-tailed deer)	United States of America	Wild Free-Living	1	no	Dermatitis	Ulcer, hyperkeratosis, crusts	Ears	NA	Louse	Alopecia, Columbian black-tailed deer, Damalinia (Cervicola), Demodex odocoilei, hair loss, lice, Odocoileus hemionus columbianus, parasites, pediculosis
Health evaluation of free-ranging and captive pichis (*Zaedyus pichiy*; Mammalia, Dasypodidae), in Mendoza Province, Argentina.	Superina, M.<br>Garner, M. M.<br>Aguilar, R. F.	2009	Journal of Wildlife Diseases	*Zaedyus pichiy* (pichi)	Argentina	Wild Free-Living, Wild Captive	45 (39 free-living, 6 Wild captive)	yes	Hypersensitivity dermatitis	Microscopic lesions	Skin	NA	Environmetnal	Besnoitia, histopathology, parasitology, pathology, serology, Trypanosoma cruzi, wildlife health.
Health evaluation of free-ranging eastern bettongs (*Bettongia gaimard*i) during translocation for reintroduction in australia	Portas, Timothy<br>Fletcher, Don<br>Spratt, David<br>Reiss, Andrea<br>Holz, Peter<br>Stalder, Kathryn<br>Devlin, Joanne<br>Taylor, David<br>Dobroszczyk, David<br>Manning, Adrian D.	2014	Journal of Wildlife Diseases	*Bettongia gaimardi* (bettong)	Australia	Wild Free-living	7	yes	dermatitis	Mild to moderate alopecia, Gross lesions included alopecia, diffuse erythema, and multifocal scaling, crusting, pustules, and ulceration. rregular acanthosis, hyperkeratosis superficial pustules, and parakeratotic crusting.	both hind limbs from the hock to the inguinal area	NA	Potenitally mites, unknown sp.	Bettongia gaimardi, eastern bettong, health evaluation, herpesvirus, parasite, toxoplasmosis, translocation.
Hepatotoxicity and secondary photosensitization in a red kangaroo (*Megaleia rufus*) due to ingestion of Lantana camara	Johnson, J. H.<br>Jensen, J. M.	1998	Journal of Zoo and Wildlife Medicine	*Megaleira rufus* (Red kangaroo)*<br>[*Macropus rufus*]	United States of America	Wild Captive	2	no	Exudative dermatitis	Crusty black exudate, exudative lesions	Ear margins, eyelids, muzzle, and scrotum and opacity of the corneas	Plant Matter - (*Lantana camara*)	NA	Photosensitization, red kangaroo, Megaleia rufus, Lantana c?mara
High morbidity cutaneous enzootic myiasis by Dermatobia hominis (Diptera: *Oestridae*) in sambar deer (*Rusa unicolor)*	Pereira, A. H. B.<br>Carrão, D. L.<br>Balthazar, D. A.<br>Rocha, B. S.<br>de Araújo, J. L.<br>Caldas, S. A.<br>Ubiali, D. G.	2020	Parasitology Research	*Rusa unicolor* (sambar deer)	Brazil	Wild Captive	8	yes	Dermatitis	Eosinophilic and granulomatous chronic active severe necrohemorrhagic dermatitis	skin	Diperta - (*Dermatobia hominis*)	NA	Wildlife medicine . Oestridae . Enzootic cutaneous myiasis . Oral ivermectin . Dermatobia hominis
Histopathology and risk factors associated with *Neotrombicula microti* infestation in the endangered amargosa vole (*Microtus Californicus scirpensis*)	Ott-Conn, C. N.<br>Woods, L. W.<br>Clifford, D. L.<br>Branston, T.<br>Foley, J.	2015	Journal of Wildlife Diseases	*Microtus californicus scirpensis* (Amargosa vole)	United States of America	Wild Free-living	22	yes	Auricular dermatitis	Severe granulocytic and necrotizing dermatitis	ears, genitalia, chin	Mite - (*Neotrombicula microti*)	NA	Amargosa vole, auricular dermatitis, chiggers, Microtus californicus scirpensis, Neotrombicula microti, trombiculiasis.
Histopathologic studies on mucosal and cutaneous lesions in contagious papular dermatitis of Japanese serow (*Capricornis crispus*)	Okada, Hm<br>Okada, K<br>Numakunai, S<br>Ohshima, K	1984	The Japanese Journal of Veterinary Science	*Capricornis crispus* (Japanese serow)	Japan	Wild Captive, Wild Free-Living	14 (7 wild captive, 7 free-living)	no	Contagious papular dermatitis	Papular lesions, necrosis, scabs	Lips, muzzle, face, eyelids, udder, feet	Virus - (parapoxvirus)	NA	contagious papular dermatitis, Japanese serow
Hyperplastic dermatitis associated with acariasis in a siamang (*Symphalangus syndactylus*)	Atkins, Adrienne<br>Heard, Darryl J<br>Mertins, James W<br>Kimbro, Jason<br>Greiner, Ellis C	2008	Journal of zoo and wildlife medicine	*Symphalangus syndactylus* (siamang)	United States of America	Wild Captive	2	no	Hyperplastic dermatitis	Mange, pruritus, patchy alopecia, and scaly thickened skin	Face, abdomen, arms	Mite (*Psorobia spp.*)	NA.	Hylobates syndactylus, Symphalangus syndactylus, Psorergates cercopitheci, psorergatic mange, Psorobia, siamang

Identifying maintenance hosts for infection with *Dichelobacter nodosus* in free-ranging wild ruminants in Switzerland: A prevalence study	Moore-Jones, Gaia<br>Ardueser, Flurin<br>Duerr, Salome<br>Brawand, Stefanie Gobeli<br>Steiner, Adrian<br>Zanolari, Patrik<br>Ryser-Degiorgis, Marie-Pierre	2020	Plos One	*Capra ibex ibex* (Alpine ibex)	Switzerland	Wild Free-living	1	yes	Dermatitis	lesions, ulcerative interdigital pododermatitis	hind feet	Bacteria - (*Dichelobacter nodosus*)	NA	NA
Immune-mediated interface dermatitis in a rhesus macaque	Makaron, L.<br>Smith, K.<br>Bailey, C.<br>Kaliyaperumal, S.<br>Miller, A.<br>Kramer, J.	2012	Journal of Medical Primatology	*Macaca mulatta* (Rhesus macaques)	United States of America	Wild Laboratory	1	no	Dermatitis	palmoplantar hyperkeratosis, deep ulcers, fissures	hands and feet, axillary regions	NA	Psychogenic	NA
Inguinal and axillary dermatitis in wallabies in north Queensland due to the dermanyssid mite *Thadeua serrata*	Skerratt, Lf<br>Beveridge, I<br>Johnson, Pm	2007	Australian veterinary journal	*Petrogale persephone* (Proserpine rock wallaby), *Macropus dorsalis* (black-striped wallaby)	Australia	Wild Free-Living	9 (6 rock wallabies, 3 black-striped wallabies)	no	Dermatitis	Lesions, hyperkeratotic epidermis	Inguinal and axillary skin	mite - (*Thadeua serrata*)	NA	NA
Isolation of digital dermatitis treponemes from hoof lesions in wild North American elk (*Cervus elaphus*) in Washington State, USA	Clegg, S. R.<br>Mansfield, K. G.<br>Newbrook, K.<br>Sullivan, L. E.<br>Blowey, R. W.<br>Carter, S. D.<br>Evans, N. J.	2015	Journal of Clinical Microbiology	*Cervus elaphus* (American elk)	United States of America	Wild Free-Living	7	no	Digital dermatitis	Grossly, affected elk have deformed hooves that are asymmetrical, markedly elongated, and curved or broken or with sloughed horn. Erosive lesions at the coronary band, underrun horn of the wall and sole	Hooves/elk feet	Bacteria (*Treponema spp* .)	NA.	NA
Isolation of *Staphylococcus simulans* from dermatitis in a captive African pygmy hedgehog	Han, Jae-Ik<br>Lee, Sook-Jin<br>Jang, Hye-Jin<br>Kim, Jeong-Ho<br>Na, Ki-Jeong	2011	Journal of zoo and wildlife medicine	*Erinaceus albiventris* (African pygmy hedgehog)	United States of America	Wild Captive	1	no	Dermatitis	Hyperkeratosis, alopecia, lesions. erythema, exudation, and pinpoint hemorrhages were observed in the lesion	Back of hedgehog	NA	Bacteria - (*Staphylococcus simulans*)	Alopecia, dermatitis, hedgehog, 16S rRNA gene, Staphylococcus simulans.
Leprosy in red squirrels in Scotland	Meredith, Anna<br>Del Pozo, Jorge<br>Smith, Sionagh<br>Milne, Elspeth<br>Stevenson, Karen<br>Mcluckie, Joyce	2014	Veterinary Record	*Sciurus vulgaris* (red squirrels)	Scotland	Wild Free-Living	6	yes	Dermatitis	Alopecia, cutaneous swelling, granulomatous dermatitis	Snout, lips, eyelids, ponnae, distal aspect of limbs	Bacteria - (*Mycobacterium lepromatosis*)	NA	NA
Lesions associated with infestation of a yellow-footed rock wallaby (*Petrogale xanthopus xanthopus*) with larvae of *Odontacarus (Leogonius) adelaideae* (Womersley)(Acarina: Trombiculidae) in South Australia	O'Callaghan, Michael G<br>Carmichael, Ian H<br>Finnie, John W<br>Conaghty, Sue	1994	Journal of Wildlife Diseases	*Petrogale xanthopus xanthopus* (yellow-footed rock wallaby)	Australia	Wild Free-Living	1	no	Pustular dermatitis	Extensive, moist, pustular dermatitis, hyperkeratosis epidermis	Inguinal, abdominal and axiltary regions.	Mite - (*Odontacarus adelaideae*)	NA	Odon taca rus adelaideae, Trombiculidae, Leeuwenhoekiinae, chiggers, dermatitis, yellow-footed rock wallaby, Petrogale xanthopus xanthopus.
Malassezia pachydermatis isolated from a South American sea lion (*Otaria byronia*) with dermatitis	Nakagaki, Kazuhide<br>Hata, Kunihiko<br>Iwata, Eri<br>Takeo, Kanji	2000	Journal of Veterinary Medical Science	*Otaria byronia* (South American sea lion)	Japan	Wild Captive	1	no	Dermatitis	Cutaneous specimens	NA	Fungus (*Malassezia pachydermatis*)	NA	Malassezia pachydermatis, Otaria byronia, protoplasmic membrane, ultrastructure
Marine debris in harbour porpoises and seals from German waters	Unger, B.<br>Herr, H.<br>Benke, H.<br>Boehmert, M.<br>Burkhardt-Holm, P.<br>Daehne, M.<br>Hillmann, M.<br>Wolff-Schmidt, K.<br>Wohlsein, P.<br>Siebert, U.	2017	Marine Environmental Research	*Phoca vitulina* (harbour seal), *Halichoerus grypus* (grey seal)	Germany	Wild Free-living	5 [3 *Phoca vitulina* (harbour seal);<br>2 *Halichoerus grypus* (grey seal)]	yes	Ulcerative dermatitis	suppurative ulcerative dermatitis, lesions	neck	NA	Sea debris - intralesional marine debris indicating that the debris represents the most likely cause of the tissue damage.	Pollution effects, Pollutant burden, Environmental, impact, Fishing gear, North Sea, Baltic Sea, Harbour porpoise, Harbour seal, Grey seal, Health status
The morphology and pathology of Besnoitia sp. in reindeer *(Rangifer tarandus tarandus*)	Ayroud, M.<br>Leighton, F. A.<br>Tessaro, S. V.	1995	Journal of Wildlife Diseases	*Rangifer tarandus tarandus* (mountain reindeer)	Canada	Wild Captive	4	no	Dermatitis	alopecia, mild thickening anf encrustation of skin.	carpal, tarsal	Apicomplexan - (*Besnoitia sp.*)	NA	Reindeer, Rangifer tarandus tarandus, Besnoitia, protozoa, pathology, ultrastructure,<br>life cycle, cat.
Molecular detection of pseudogymnoascus destructans (Ascomycota: Pseudeurotiaceae) and unidentified fungal dermatitides on big brown bats *(Eptesicus fuscus*) overwintering inside buildings in Canada	McAlpine, D. F.<br>McBurney, S.<br>Sabine, M.<br>Vanderwolf, K. J.<br>Park, A.<br>Cai, H. Y.	2016	Journal of Wildlife Diseases	*Eptesicus fuscus* (big brown bats)	Canada	Wild Free-living	2	yes	Dermatitis	mild, focal,<br>pustular, fungal dermatitis, lesions	muzzle	NA	unidentified ascomycetes.	Big brown bat, Eptesicus fuscus, fungal infection, Pseudogymnoascus destructans,<br>white-nose syndrome.
Mortalities, amyloidosis and other diseases in free-living red squirrels (*Sciurus vulgaris*) on Jersey, Channel Islands	Blackett, T. A.<br>Simpson, V. R.<br>Haugland, S.<br>Everest, D. J.<br>Muir, C. F.<br>Smith, K. C.<br>Mill, A. C.	2018	Veterinary record	*Sciurus vulgaris* (red squirrels)	Bailiwick of Jersey	Wild Free-Living	49	yes	Exudative dermatitis	Lesions morphologically consistent with those of exudative, pustular dermatitis accompanying alopecia and sloughing of the digital and/or metacarpal pads with necrosis of the digits	Gross lesions were typically seen around the mouth, nose and on the feet occasionally on the eyelids and more infrequently on the scrotum and pinnae	NA	Bacetria - (*Staphylococcus aureus*)	disease surveillance; histopathology; squirrels
Mortality in red squirrels (*Sciurus vulgaris*) associated with exudative dermatitis	Simpson, V. R.<br>Hargreaves, J.<br>Everest, D. J.<br>Baker, A. S.<br>Booth, P. A.<br>Butler, H. M.<br>Blackett, T.	2010	Veterinary record	*Sciurus vulgaris* (red squirrels)	England, Bailiwick of Jersey	Wild Free-Living	11	yes	Exudative dermatitis	Exudative, ulcerative dermatitis and superficial staphylococcal pyoderma, gross lesions, dry scabby lesions	Lips, eyelids and feet	NA	NA	NA
Mycotic dermatitis in a vagrant parti-coloured bat (*Vespertilio murinus*) in Great Britain	Barlow, Alex<br>Jolliffe, Tracey<br>Tomlin, Mike<br>Worledge, Lisa<br>Miller, Helen	2011	Veterinary Record	*Vespertilio murinus* (parti-coloured bat)	Scotland	Wild Free-Living	1	yes	Necrotising dermatitis	Several small white lesions were seen on the wing membranes. Acute focal necrotising dermatitis with intralesional fungal elementsamorphous eosinophilic material permeated by fungal hyphae and fruiting bodies associated with a marginal band of basophilic leucocytoclastic debri	Wing membrane	Fungus - (*Cladosporium spp* ; and *Rhodotorula spp* .)	NA	NA
Mycotic dermatitis in an Atlantic white-sided dolphin, a pygmy sperm whale, and two harbor seals	Frasca, Jr S<br>Dunn, Jl<br>Cooke, Jc<br>Buck, Jd	1996	Journal of the American Veterinary Medical Association	*Phoca vitulina* (harbor seal)	United States of America	Wild Free-Living	2	no	Mycotic dermatitis	Raised, firm, erythematous, cutaneous nodules	Heads, trunks, caudal portions	Fungus - (*Fusarium spp* .)	NA	NA
Naturally occurring cutaneous streptothricosis in a cottontail rabbit	Shotts, Emmett Booker<br>Kistner, Theodore P	1970	Journal of the American Veterinary Medical Association	*Sylvilagus floridanus* (cottontail rabbit)	United States of America	Wild Free-Living	2	no	Mycotic dermatitis	Lesions, dry purulent material, irritated skin, fibrinopurulent exudate, neucrotic debris	Feet, footpads, leg, anus and urethra, muzzle	Bacteria - (*Dermatophilus congolensis*)	NA	NA
New records of hair follicle mites (Demodecidae) from North American Cervidae	Desch, Clifford E<br>Andrews, John J<br>Baeten, Laurie A<br>Holder, Zach<br>Powers, Jenny G<br>Weber, Duane<br>Ballweber, Lora R	2010	Journal of Wildlife Diseases	*Odocoileus hemionus* (mule deer), *Cervus elaphus nelsoni* (Rocky Mountain elk), *Odocoileus virginianus* (white-tailed deer)	United States of America	Wild Free-Living	3 (1 of each study species)	no	Nodular dermatitis	Alopecia, lesions, inflamation, nodular dermatitis	Abdomen, hind quarters, shoulders, carpi, hocks,	Mite - (*Demodex kutzeri*)	NA	Cervus elaphus, Demodex kutzeri, Demodex odocoilei, ectoparasites, host specificity, Odocoileus hemionus, Odocoileus virginianus, synhospitalic.
Notoedric mange in gray squirrels (*Sciurus carolinensis*)	Carlson, Bl<br>Roher, Dp<br>Nielsen, Sw	1982	Journal of Wildlife Diseases	*Sciurus carolinensis* (gray squirrel)	United States of America	Wild Free-Living	7	no	Dermatitis	Alopecia, skin thicken and wrinkled, thick yellow crusts	Head, neck, upper torso, tail	Mite - (*Notoedres sp.*)	NA	NA
A novel poxvirus lethal to red squirrels (*Sciurus vulgaris*)	Thomas, Kathryn<br>Tompkins, Daniel M<br>Sainsbury, Anthony W<br>Wood, Ann R<br>Dalziel, Robert<br>Nettleton, Peter F<br>Mcinnes, Colin J	2003	Journal of General Virology	*Sciurus vulgaris* (red squirrels)	England	Wild Laboratory	2	yes	Dermatitis	Severe secondary<br>lesions, scabs	Face, feet, hands	Virus - (parapoxvirus)	NA	NA
Observations of a denning-related dermatitis in American black bears	Costello, Cecily M<br>Quigley, Kathy S<br>Jones, Donald E<br>Inman, Robert M<br>Inman, Kristine H	2006	Ursus	*Ursus americanus* (American black bear)	United States of America	Wild Free-Living	85	no	Dermatitis	Undiagnosed dermatitis, alopecia and edema. Affected areas appeared smooth and red, particularly the eyelids	Head, neck, and thorax. Dermatitis was confined to the area around the eyes and down the muzzle	NA	NA	alopecia, American black bear, denning, dermatitis, hibernation, mange, New Mexico, Ursus americanus
Occurrence of Demodex Owen 1843 on a white-tailed deer from Oklahoma	Carpenter, James W<br>Freeny, J Clay<br>Patton, Clark S	1972	Journal of Wildlife Diseases	*Odocoileus virginianus* (white-tailed deer)	United States of America	Wild Free-Living	1	no	Dermatitis	Alopecia	Abdomen, rump, thighs	Mite - (*Demodex sp.*)	NA	NA
Oral disease in free-living red squirrels (*Sciurus vulgaris*) in the United Kingdom	Sainsbury, Anthony W<br>Kountouri, Amalia<br>Duboulay, George<br>Kertesz, Peter	2004	Journal of Wildlife Diseases	*Sciurus vulgaris* (red squirrels)	England	Wild Free-Living	1	yes	Exudative erythematous dermatitis	Skin lesions	Face	Virus - (parapoxvirus)	NA	Incisors, malocclusion, molars, periodontal disease, rodent, rodentia.

Oral, nasal, and cutaneous eosinophilic granulomas in the black rhinoceros (*Diceros bicornis*): a lesion distinct from superficial necrolytic dermatitis	Pessier, Allan P<br>Munson, Linda<br>Miller, R Eric	2004	Journal of Zoo and Wildlife Medicine	*Diceros bicornis michaeli* (black rhinoceros, eastern sub-species)	United States of America	Wild Captive	8	no	Superficial necrolytic dermatitis	Lesions, oral bleeding or epistaxis from multilobulated, fungating, proliferative masses with areas of ulceration. mucosal or epidermal hyperplasia. noceroses with cutaneous, oral, or nasal mucosal ulcerative lesion	Oral, nasal, and cutaneous lesions. occurred most often behind the prehensile lip	NA	Classed as EG (eosinophilic granulomas), but could be nematodes, or could be fungal.	Black rhinoceros, Diceros bicornis, eosinophilic granuloma, superficial necrolytic dermatitis, ulcerative dermatopathy.
Orf virus infection in Alaskan mountain goats, Dall's sheep, muskoxen, caribou and Sitka black-tailed deer	Tryland, Morten<br>Beckmen, Kimberlee Beth<br>Burek-Huntington, Kathleen Ann<br>Breines, Eva Marie<br>Klein, Joern	2018	Acta veterinaria scandinavica	*Oreamnos americanus* (mountain goat), *Ovis dalli dalli* (dalls sheep), *Ovibos moschatus* (Muskoxen), *Odocoileus hemionus sitkensis* (Sitka black-tailed deer), *Rangifer tarandus granti* (caribou)	United States of America	Wild Free-Living	16 (8 goat, 3 sheep, 3 ox, 1 deer, 1 caribou)	no	Ulcerative and proliferative dermatitis	Proliferative massessm dark crusts, ulcerative lesions, ulcers	Palpebrae, nares, lips, anus, prepuce or vulva, as well as coronary bands, muzzel, eyes, ears	Virus - (parapoxvirus)	NA	Alaska, Caribou, Contagious ecthyma, Dall's sheep, Deer, Mountain goat, Muskox, Parapoxvirus, Virology, Wildlife, Zoonosis
An outbreak of Caparinia tripilis in a colony of African pygmy hedgehogs (*Atelerix albiventris*) from Korea	Kim, D. H.<br>Oh, D. S.<br>Ahn, K. S.<br>Shin, S. S.	2012	Korean Journal of Parasitology	*Atelerix albiventris* (African hedghog)	Korea	Wild Captive	22	no	dermatitis	scale, self-trauma, puritis, flaking, crusts, scabs	mantle armpit, ear	Mite - (*Caparinia tripilis*)	NA	Caparinia tripilis, hedgehogs, Atelerix albiventris, case report
Palmoplantar nonpustular psoriasiform dermatitis in a rhesus macaque	Piedras, María José GM<br>García-Cabezas, Miguel Ángel<br>Sendagorta, Elena<br>Miró-Murillo, Marta<br>Cavada, Carmen	2011	Veterinary dermatology	*Macaca mulatta* (Rhesus macaques)	Spain	Wild Laboratory	1	no	psoriasiform dermatitis	palmoplantar nonpustular psoriasis, scaly plaques, nail hyperkeratosis, epidermal hyperkeratosis with multifocal parakeratosis, Erythematous and scaly coalescent plaques	palms, soles, nails	NA	NA	NA
Parapoxvirus causes a deleterious disease in red squirrels associated with UK population declines	Tompkins, Daniel M<br>Sainsbury, Anthony W<br>Nettleton, Peter<br>Buxton, D<br>Gurnell, J	2002	Proceedings of the Royal Society of London. Series B: Biological Sciences	*Sciurus vulgaris* (red squirrels)	England	Wild Free-Living, Wild Laboratory	10 (6 wild free-living, 4 wild labortaory red squirrels)	yes	Exudative erythematous dermatitis	Skin lesions, secondary lesions	Face, ventral skin surfaces of the feet and body, medial skin of the legs and the genital region	Virus - (parapoxvirus)	NA	ecological replacement; emerging infectious disease; grey squirrel; Sciurus carolinensis; Sciurus vulgaris; virus
Parapoxvirus infections in New Zealand farmed red deer (*Cervus elaphus*)	Horner, GW<br>Robinson, AJ<br>Hunter, R<br>Cox, BT<br>Smith, R	1987	New Zealand Veterinary Journal	*Cervus elaphus* (red deer)	New Zealand	Wild Captive	498	no	Proliferative dermatitis	Scabby lesions, alopecia, crusting, proliferative viral dermatitis. Hyperplasia, dyskeratosis, parakeratosis	muzzle, lips, face, ears, neck, legs, eyelids, mouth, eyelids, perineum,	Virus - (orf - parapoxvirus)	NA	NA
Parapoxvirus infections of red deer, Italy	Scagliarini, Alessandra<br>Vaccari, Francesca<br>Turrini, Filippo<br>Bianchi, Alessandro<br>Cordioli, Paolo<br>Lavazza, Antonio	2011	Emerging infectious diseases	*Cervus elaphus* (red deer)	Italy	Wild Free-Living	1	no	Contagious pustular dermatitis	Proliferative lesions, erosions, and ulcers	Lips, hard palate	Virus - (parapoxvirus)	NA	Red deer, Chordopoxvirinae, parapoxvirus, evolution, zoonoses, viruses, Italy, dispatch
Parasites and associated pathology of the swamp wallaby, *Wallabia bicolor* (Marsupialia)	Beveridge, I<br>Presidente, PJA<br>Speare, R	1985	Journal of Wildlife Diseases	*Wallabia bicolor* (swamp wallaby)	Australia	Wild Free-living	35	no	dermatitis	mild localized dermatitis	NA	Tick - (*Amblyomrrw triguttatum* & *Haemaphysalis bancrofti*)	NA	NA
Parasites of the brush-tailed rock-wallaby (*Petrogale penicillata*)	Barnes, Tamsin S<br>Goldizen, Anne W<br>Morton, John M<br>Coleman, Glen T	1985	Journal of Wildlife Diseases	*Petrogale penicillata* (brush-tailed rock-wallaby)	Australia	Wild Free-Living	88	yes	Localized dermatitis	Skin lesions, granuloma, mild localized dermatitis	Ear, neck, eyelids, inguinal area, axillary area, shoulders, thighs, chest, pouch, base of tail, flank, genitalia, nose, mandible, and elbow	Tick - (*IIxodes holocyclus, Haemaphysalis bancrofti*)	NA	Brush-tailed rock-wallaby, coccidia, ectoparasite, fecal egg count, macropodid, Petrogale penicillata, Toxoplasma gondii.
Pathogen prevalance in American black bears (*Ursus Americanus amblyceps*) of the Jemez Mountains, New Mexico, USA	Bard, S. M.<br>Cain, J. W., Iii	2019	Journal of Wildlife Diseases	*Ursus americanus amblyceps* (New Mexico black bear)	United States of America	Wild Free-Living	5	yes	Dermatitis	Dermatitis, presumed mange	NA	NA	Ectoparasites	Black bear, New Mexico, pathology, plague, serology, Ursus americanus, wildlife disease.
Pathologic findings in Western gray squirrels (*Sciurus griseus*) from a notoedric mange epidemic in the San Bernardino Mountains, California	Stephenson, N.<br>Swift, P.<br>Villepique, J. T.<br>Clifford, D. L.<br>Nyaoke, A.<br>De la Mora, A.<br>Moore, J.<br>Foley, J.	2013	International Journal for Parasitology: Parasites and Wildlife	*sciurus griseus* (western gray squirrel)	United States of America	Wild Free-living	5	yes	Dermatitis	parakeratotic hyperkeratosis with serocellular crusting, intracorneal pustules, severe dermatitis, lesions, mange	skin of the head (including pinnae), neck, front legs and thorax, caudoventral abdomen and dorsomedial thighs	Mite - (*Notoedres centrifera*)	NA	Dermatitis, Internal transcribed spacer ITS-2, *Notoedres centrifera*, Sciurid
Pathological and electron microscopical observations on naturally occurring contagious ecthyma outbreak in two wild goats (*Capra aegagrus aegragus*)	Ozmen, O.<br>Dolu, H.	2018	Ankara Universitesi Veteriner Fakultesi Dergisi	*Capra aegagrus aegragus* (wild goat)	Turkey	Wild Free-Living	15	yes	Contagious dermatitis	Lesions mainly localized on lips and numerous proliferative papules and pustules, necrotic scabs and swelling, as well as edema, hyperemia and ulcer. multifocal-coalescing necrotizing and proliferative cheilitis and dermatitis in a wild goat	Lips, eyelids, ears, and nose	Virus - (parapoxvirus)	NA	Contagious Ecthyma, electron microscopy, pathology, wild goats (Capra aegagrus aegragus).
Pathological findings in a captive colony of maras (*Dolichotis patagonum*)	Rosas-Rosas, A. G.<br>Juan-Sallés, C.<br>Garner, M. M.	2006	Veterinary Record	*Dolichotis patagonum* (maras)	Mexico	Wild Captive	18 - 15 (fleas); 3(other/NA)	no	Pododermatitis; ectoparasite dermatitis	Chronic unilateral pododermatitis associated with rupture of the tarsal joint capsule.<br><br>cutaneous lesions, multifocal hypotrichosis or alopecia in all seven, ulceration in five, and/or crusting, multifocal hypotrichosis or alopecia, and in<br>one there was hyperkeratosis and crusting	Paws/feet, skin	NA;<br><br>Fleas- (*Echidnophaga spp*)	Bacteria and/or Fleas (Aerobic culture from this exudate yielded *Staphylococcus, Streptococcus* and *Pseudomonas* species).<br>Seven (from 15) animals which showed flea hyperplastic dermatitis had no fleas on them, however it was still suceptected Fleas where the agent of the dermatitis	NA
Pathological Findings in Wild Rats (*Rattus rattus*) Captured at Guafo Island, Northern Chilean Patagonia	Seguel, M.<br>Munoz, F.<br>Paredes, E.<br>Navarrete, M. J.<br>Gottdenker, N. L.	2017	Journal of Comparative Pathology	*Rattus rattus* (black rat)	Chile	Wild Free-Living	105	yes	Dermatitis	Lesions, coalescing areas of yellow ro orange crusting sometimes forming nodular areas of alopecia, Lymphoplasmacytic and eosinophilic hyperkeratotic	Random distribution on body	Mite - (*Ornithonyssus bacoti*)	NA	black rat, Guafo Island, pathogens, pathology
Pathology and distribution of trombiculosis in northern chamois (*Rupicapra rupicapra rupicapra)* in the Italian Alps	Salvadori, C.<br>Formenti, N.<br>Trogu, T.<br>Lanfranchi, P.<br>Rossi, L.<br>Citterio, C.<br>Obber, F.<br>Poli, A.	2019	Journal of Wildlife Diseases	*Rupicapra rupicapra* (chamois)	Italy	Wild Free-living	40	no	Dermatitis	focal moderate dermatitis with epidermal necrosis, thin crusts, and hyperkeratosis, lesions	muzzle, head (pinnae and areas around eyes and mouth) and limbs	Mite - (family Trombiculidae)	NA	Chiggers, dermatitis, histopathology, larval mites, northern chamois, Rupicapra rupicapra rupicapra, Trombiculidae, trombiculosis.
Pathology of interdigital glands in a wild Japanese serow (*Capricornis crispus*) infected with parapoxvirus	Suzuki, Yoshitaka<br>Komatsu, Takeshi<br>Yamamoto, Yoshio<br>Atoji, Yasuro	1997	Journal of veterinary medical science	*Capricornis crispus* (Japanese serow)	Japan	Wild Free-Living	1	no	Contagious papular dermatitis	Lesions, hyperkeratosis	Interdigital glands, feet	Virus - (parapoxvirus)	NA	interdigital gland, Japanese serow, parapoxvirus
Pathology of sarcoptic mange in red foxes (*vulpes vulpes*): Macroscopic and histologic characterization of three disease stages	Nimmervoll, H.<br>Hoby, S.<br>Robert, N.<br>Lommano, E.<br>Welle, M.<br>Ryser-Degiorgis, M. P.	2013	Journal of Wildlife Diseases	*Vulpes vulpes* (red fox)	Switzerland	Wild Free-living	150	yes	mange dermatitis	skin lesion, cursts, hyperpigmentation, local dermatitis	body	Mite - (*Sarcoptes scabiei*)	NA	Classification, dermatitis, disease stage, histopathology, mange, red fox, *Sarcoptes scabiei*, *Vulpes vulpes*
Pelodera strongyloides infection in Pacific harbor seals (*Phoca vitulina richardi* i) from California	McHuron, Elizabeth A.<br>Miller, Melissa A.<br>Gardiner, Chris H.<br>Batac, Francesca I.<br>Harvey, James T.	2013	Journal of Zoo and Wildlife Medicine	*Phoca vitulina* (harbor seal)	United States of America	Wild Free-living	4	no	Dermatitis	Mild chronic superficial dermatitis and perifolliculitis	face and head, back, dorsal sides, and around its flippers	Nematode - (*Pelodera strongyloides*)	NA	Dermatitis, harbor seal, Pelodera strongyloides, Phoca vitulina, Tomales Bay.
Pen trial of estrogen-induced conditioned food aversion to eggs in raccoons (*Procyon lotor*)	Dueser, Raymond D.<br>Martin, Joel D.<br>Moncrief, Nancy D.	2018	Applied Animal Behaviour Science	*Procyon lotor* (raccoons)	United States of America	Wild Laboratory	7	yes	dermatitis	dermatitis and patchy hair	NA	2 Fungus - dermatophytosis ;<br><br>5 unknown	2 dermatophilosis of some kind, other 5 are unknown, and potentially caused by the treatment of estrogen.	Behavior, Mesopredator, Conditioned taste aversion, Deception-based food aversion, 17 α-ethinyl estradiol, Egg predation

Phoma (*peyronellaea*) as zoopathogen	Gordon, M. A.<br>Salkin, I. F.<br>Stone, W. B.	1975	Sabouraudia, Journal of Medical and Veterinary Mycology	*Odocoileus virginianus* (white-tailed deer)	United States of America	Wild Free-living	1	no	aural dermatitis	cutaneous lesions, Severe hair loss was observed, massive epidermal scaling was noted on a large portion of the ears.	ears, sides of the face and neck	Fungus - (*Phoma cava*)	NA	NA
Pododermatitis in farmed mink in Canada	Brojer, Caroline	2000	The University of Guelph/National Library of Canada - Thesis	*Neovison vison* (Mink)	Canada	Wild Captive	246	no	Pododermatitis	ulcerated, cmsty areas of thickened skin. Alopecia, ulcers, crusts, hyperkeratosis, lesions, necrosis, footpad dermatitis	foot, face, nose, eyelids, head	NA	Potentially agent was a contaminant on seal meat	NA
Pododermatitis in farmed mink *(Neovison vison*) in Spain	Fernández-Antonio, R<br>Fraílde, LD<br>Losada, AP<br>López-Peña, M<br>Vázquez, S<br>Bermúdez, R<br>Quiroga, MI<br>Nieto, JM	2008	IX International Scientific Congress in Fur Animal Production	*Neovison vison* (Mink)	Spain	Wild Captive	3	no	Pododermatitis	ulcers, scabs, debris, lesions, hyperkeratosis, necrosis, crusting and abscesses.	foot pads, face	NA	Potential bacterial infection	NA
Poxvirus identified in a red squirrel (*Sciurus vulgaris*) from Spain	Obon, Elena<br>Juan-Sallés, Carles<br>McInnes, Colin J.<br>Everest, David J.	2011	Veterinary Record	*Sciurus vulgaris* (red squirrels)	Spain	Wild Free-living	1	no	Dermatitis	skin lesions, exudative, ulcerative dermatitis with crusts	ear tips, tail, feet	Virus - (Poxvirus family)	NA	NA
Prevalence and spatio-temporal variation of an alopecia syndrome in polar bears (*Ursus maritimus*) of the southern Beaufort Sea	Atwood, Todd<br>Peacock, Elizabeth<br>Burek-Huntington, Kathy<br>Shearn-Bochsler, Valerie<br>Bodenstein, Barbara<br>Beckmen, Kimberlee<br>Durner, George	2015	Journal of Wildlife Diseases	*Ursus maritimus* (polar Bear)	United States of America	Wild Free-living	49	yes	chronic proliferative dermatitis	alopecia, exudation and crusted skin lesions	head, neck, shoulders	NA	NA	Alopecia, Arctic, *Ursus maritimus*, disease, polar bears, skin lesion.
A previously unidentified Chorioptes species infesting outer ear canals of moose (*Alces alces*): characterization of the mite and the pathology of infestation	Hestvik, Gete<br>Zahler-Rinder, Monika<br>Gavier-Widén, Dolores<br>Lindberg, Ronny<br>Mattsson, Roland<br>Morrison, David<br>Bornstein, Set	2007	Acta veterinaria Scandinavica	*Alces alces* (Moose)	Sweden	Wild Free-living	43	no	Dermatitis	lesions, crusts, perivascular to interstitial dermatitis with epidermal hyperkeratosis	ears, ear canal	Mite - (*Chorioptes* sp.)	NA	NA
Psorergatic acariasis in vervet monkeys	Seier, Jv	1985	Laboratory animals	*Cercopithecus pygerythrus* (vervet monkeys)	South Africa	Wild Laboratory	7	no	Dermatitis	Lesions, alopecia	Eyelids	Mite - (*Psorergates cercopitheci*)	NA	Cercopithecus; Mite infestations
Psoroptic scabies in desert bighorn sheep (*Ovis canadensis nelsoni*) from northwestern Arizona	Welsh, George W<br>Bunch, Thomas D	1983	Journal of Wildlife Diseases	*Ovis canadensis nelsoni* (bighorn sheep)	United States of America	Wild Free-Living	5	yes	Psoroptic scabies dermatitis	Red, crusted epidermis	Ears	Mite - (*Psoroptes ovis*)	NA	NA
Purulent Trunk Dermatitis in a Male Ceylon Elephant (*Elephas maximus maximus*)	Vodička, R	2008	Acta Veterinaria Brno	*Elephas maximus* (Asian Elephant)	Czechia	Wild Captive	1	no	Purulent dermatitis	purulent trunk dermatitis, skin lesions, pus,	trunk, tail, behind the ears	NA	Probably insufficient humidification, potentially caused by bacteria isolated: *Staphylococcus spp.*, *Streptococcus spp.* and *Candida tropicalis.*	Proboscidea, purulent dermatitis, anaesthesia, haematology, biochemistry
Pustular dermatitis caused by impetigo in red-tailed monkeys (*Cercopithecus ascanius*)	Coughlin, Patrick<br>Bradford, Carol<br>Montali, Richard J<br>Bronson, Ellen	2018	Journal of Zoo and Wildlife Medicine	*Cercopithecus ascanius* (red-tailed monkey)	United States of America	Wild Captive	2	no	Pustular dermatitis	scabbing and ulceration, suppurative, necrotizing, pustular cheilitis, pustular dermatitis, multifocal vasculitis	philtrum, nostrils and nasal planum, pinnae, tongue, nose	Bacteria - (*Staphylococcus sp.*)	NA	Cercopithecus ascanius, Staphylococcus, dermatitis, impetigo, red-tailed monkey.
Red squirrels in the British Isles are infected with leprosy bacilli	Avanzi, Charlotte<br>Del-Pozo, Jorge<br>Benjak, Andrej<br>Stevenson, Karen<br>Simpson, Victor R<br>Busso, Philippe<br>Mcluckie, Joyce<br>Loiseau, Chloé<br>Lawton, Colin<br>Schoening, Janne	2016	Science	*Sciurus vulgaris* (red squirrels)	Scotland	Wild Free-Living	4	yes	Granulomatous dermatitis	Lesions, alopecia, extensive swelling	Snout, lips, eyelids, ear pinnae, and limb extremities	Bacteria - (*Mycobacterium lepromatosis*)	NA	NA
Regional alopecia and dermatitis due to *Lodderomyces elongisporus* in a North American porcupine (*Erethizon dorsatum*)	St Clair, Laura<br>Hopf, Cynthia<br>Peters-Kennedy, Jeanine<br>Mazulis, Christina<br>Miller, Julia<br>Scott, Danny W<br>Childs-Sanford, Sara	2020	Veterinary dermatology	*Erethizon dorsatum* (North American porcupines)	United States of America	Wild Free-living	1	no	Dermatitis	alopecia and scaling dermatitis, mild eosinophilic perivascular-to-interstitial dermatitis, alopecia, hyperpigmentation	caudal dorsum,	Fungus - (*Lodderomyces elongisporus*)	NA	NA
Responses of red foxes to first and second infection with *Sarcoptes scabiei*	Little, Susan E.<br>Davidson, William R.<br>Rakich, Pauline M.<br>Nixon, Tammy L.<br>Bounous, Denise I.<br>Nettles, Victor F.	1998	Journal of Wildlife Diseases	*Vulpes vulpes* (red fox)	United States of America	Wild Laboratory	1	no	Dermatitis	vere scrotal dermatitis<br>and associated bilateral testicular atrophy	scrotum	Mite - (*Sarcoptes scabiei*)	NA	Hypersensitivity, immune response, red fox, Sarcoptes scabiei, sarcoptic mange,<br>Vulpes vulpes.
Rhinoceros feet step out of a rule-of-thumb: A wildlife imaging pioneering approach of synchronized computed tomography-digital radiography	Galateanu, G.<br>Hermes, R.<br>Saragusty, J.<br>Göritz, F.<br>Potier, R.<br>Mulot, B.<br>Maillot, A.<br>Etienne, P.<br>Bernardino, R.<br>Fernandes, T.<br>Mews, J.<br>Hildebrandt, T. B.	2014	Plos One	*Ceratotherium simum simum* (southern white rhinoceros); *Rhinoceros unicornis* (Indian Rhinoceros)	France	Wild Captive	2 [1 - (southern white rhinoceros); 1- (Indian Rhinoceros)]	yes	Dermatitis/ pododermatitis	Ceratotherium simum simum (southern white rhinoceros) - generalized chronic ulcerative dermatitis.<br><br>Rhinoceros unicornis (Indian Rhinoceros - chronic pododermatitis.	NA/four limbs	NA	NA	NA
The role of host and environmental factors in the epidemiology of rumpwear in brushtail possums	Hufschmid, J.<br>Handasyde, K. A.<br>Beveridge, I.	2010	Australian Journal of Zoology	*Trichosurus cunninghami* (mountain brushtail possum);*Trichosurus vulpecula* (common brushtail possum)	Australia	Wild Free-Living	131<br>(114 *T. cunninghami* and 17 *T. vulpecula*)	no	Exudative dermatitis	Coat damage in the lumbo-sacral region. Mild to<br>severe hair damage (breakage of tips and matting), alopecia,<br>varying degrees of dermatitis and thickening of the skin	Coat<br>damage in the lumbo-sacral region	NA	NA	Trichosurus, body condition, environmental factors, epidemiology, fur, mechanical damage, opossums, skin lesions, New Zealand
Sarcoptic mange and Pelodera dermatitis in an American black bear (*Ursus americanus*)	Fitzgerald, Scott D<br>Cooley, Thomas M<br>Cosgrove, Melinda K	2008	Journal of Zoo and Wildlife Medicine	*Ursus americanus* (American black bear)	United States of America	Wild Free-Living	1	no	Dermatitis and mange	Hair loss, lichenification, crusting, and focal erosions, ulcerative lesions	Lateral thorax, dorsal back, face, and all four paws	Mite - (*Sarcoptes scabiei*), and Nematode - (*Pelodera strongyloides*)	NA	Black bear, dermatitis, mange, Pelodera, Sarcoptes.
Sarcoptic mange in agile wallabies (*Macropus agilis*) in the Northern Territory	Mclelland, Dj<br>Youl, Jm	2005	Australian veterinary journal	*Macropus agilis* (agile wallaby)	Australia	Wild Captive	2	yes	Pruritic dermatitis (mange)	Crusts	Hind limbs, tail, trunk, face, forelimbs	Mite - (*Sarcoptes scabiei*)	NA	NA
Sarcoptic mange in endangered kit foxes (*Vulpes macrotis mutica*): Case histories, diagnoses, and implications for conservation	Cypher, B. L.<br>Rudd, J. L.<br>Westall, T. L.<br>Woods, L. W.<br>Stephenson, N.<br>Foley, J. E.<br>Richardson, D.<br>Clifford, D. L.	2017	Journal of Wildlife Diseases	*Vulpes macrotis mutica* (San Joaquin kit fox)	United States of America	Wild Free-living	15	yes		Intense pruritus and dermatitis. alopecia, hyperkeratosis, and<br>encrustations,	face, legs, tail, thorax, abdomen	Mite - (*Sarcoptes scabiei*)	NA	California, kit fox, mites, Sarcoptes scabiei, sarcoptic mange, Vulpes macrotis mutica
Sarcoptic mange in free-ranging pampas foxes in the Gran Chaco, Bolivia	Deem, S. L.<br>Noss, A. J.<br>Cuéllar, R. L.<br>Villarroel, R.<br>Linn, M. J.<br>Forrester, D. J.	2002	Journal of Wildlife Diseases	*Pseudalopex gymnocercus* (Pampas foxes)*[*Lycalopex gymnocercus*]	Bolivia	Wild Free-ranging	19	yes	Pruritic dermatitis (mange)	lesions, alopecia and thickened, flaky skin, Moderate, chronic dermatitis with orthokeratotic hyperkeratosis	caudal dorsal trunk and tail	Mite - (*Sarcoptes scabiei*)	NA	Bolivia, Gran Chaco, mange,<br>pampas fox, Pseudalopex gymnocercus, Sarcoptes<br>scabiei.
Sarcoptic mange in free-ranging raccoon dogs (*Nyctereutes procyonoides*) in Japan	Ninomiya, Hiroyoshi<br>Ogata, Munetsugu	2005	Veterinary Dermatology	*Nyctereutes procyonoides* (Raccoon dog)	Japan	Wild Free-living	3	no	Pruritic dermatitis (mange)	skin lesions, alopecia, extensive dermatitis, corrugated, thickened and covered by greyish plaques of keratinous crusts, warty, cobblestone-like hyperkeratosis, severe malodour	ears, muzzle, eyes, elbow, thigh, neck	Mite - (*Sarcoptes scabiei*)	NA	NA
Sarcoptic mange in raccoons in Michigan	Fitzgerald, S. D.<br>Cooley, T. M.<br>Murphy, A.<br>Cosgrove, M. K.<br>King, B. A.	2004	Journal of Wildlife Diseases	*Procyon lotor* (raccoons)	United States of America	Wild Free-Living	3	no	Pruritic dermatitis (mange)	Intensely pruritic, advanced alopecic and crusting lesions, crusting and hyperkeratotic dermatitis with high numbers of sarcoptes scabiei adults, larvae, nymphs, and eggs.	Dorsum and hind limbs	Mite - (*Sarcoptes scabiei*)	NA	Parasites, pathology, Procyon<br>lotor, raccoon, Sarcoptes scabiei, sarcoptic<br>mange.

Sarcoptic mange (*Sarcoptes scabiei)* in wild canids (*Cerdocyon thous*)	Teodoro, Tamires G. W.<br>Lima, Pamela A.<br>Stehling, Patricia C.<br>Oliveira Junior, Ivam M.<br>Varaschin, Mary S.<br>Wouters, Flademir<br>Wouters, Angelica T. B.	2018	Pesquisa Veterinaria Brasileira	*Cerdocyon thous* (*crab-eating foxes*)	Brazil	Wild Free-living	2	no	Pruritic dermatitis (mange)	marked acanthosis and hyperkeratosis, and mild superficial dermatitis, extensive alopecia with thick skin crusts	head, skin, thorax, limbs and tail	Mite - (*Sarcoptes scabiei*)	NA	Sarcoptic mange, Sarcoptes scabiei, Cerdocyon thous, wild canids, skin diseases, mites, scabies, parasitoses.
Sarcoptic mange in wild carnivores and its co-occurrence with parasitic helminths in the Western Italian Alps	Balestrieri, Alessandro<br>Remonti, Luigi<br>Ferrari, Nicola<br>Ferrari, Angelo<br>Valvo, Tatiana<br>Robetto, Serena<br>Orusa, Riccardo	2006	European Journal of Wildlife Research	*Vulpes vulpes* (red fox)	Italy	Wild Free-living	32	yes	Pruritic dermatitis (mange)	severe dermatitis, lesions, pruritus, alopecia, thickened skin	half of body or more	Mite - (*Sarcoptes scabiei*)	NA	Sarcoptic mange . Red fox . Helminths . Concomitant infection
Seborrheic dermatitis in a rhesus monkey (*Macaca mulatta*)	Newcomer, Ce<br>Fox, Jg<br>Taylor, Rm<br>Smith, De	1984	Laboratory animal science	*Macaca mulatta* (Rhesus macaques)	United States of America	Wild Laboratory	1	no	Seborrheic dermatitis	Erythematous, exfoliative, pruritc, exudative skin lesions, alopecia,	Face, extremities	NA	May have been due to malnutrition (as experiments were done with withholding food)	seborrheic dermatiis, Macaca
Serum chemistry, hematologic, and post-mortem findings in free-ranging bobcats (*Lynx rufus*) with notoedric mange	Serieys, L. E.<br>Foley, J.<br>Owens, S.<br>Woods, L.<br>Boydston, E. E.<br>Lyren, L. M.<br>Poppenga, R. H.<br>Clifford, D. L.<br>Stephenson, N.<br>Rudd, J.<br>Riley, S. P.	2013	Journal of Parasitology	*Lynx rufus* (bobcat)	United States of America	Wild Free-Living	18	yes	dermatitis	severe dermatitis, acanthosis, hyperkeratosis, and parakeratosis with serocellular crusts and subcorneal pustules, alopecia, crusts, excoriations, and lichenification,	head, ears, neck, generalized	18/18 - Mite - (*Notoedres cat* i)<br><br>2/18 - Fungus (*Malassezia sp*.)	NA	NA
A severe outbreak of contagious ecthyma (orf) in a free-ranging musk ox (*Ovibos moschatus*) population in Norway	Vikøren, Turid<br>Lillehaug, Atle<br>Åkerstedt, Johan<br>Bretten, Tord<br>Haugum, Magne<br>Tryland, Morten	2008	Veterinary microbiology	*Ovibos moschatus* (muskox)	Norway	Wild Free-Living	19	no	Contagious papular dermatitis	Porliferative wart-like lesions, scabby, ulcerations	Muzzle, lips, limbs, udder, oral mucosa	Virus - (parapoxvirus)	NA	Contagious ecthyma; Musk ox; Orf; Ovibos moschatus; Parapoxvirus; Pustular dermatitis
Severe ulcerative dermatitis in platypus (*Ornithorhynchus anatinus*)	Munday, Bl<br>Peel, Bf	1983	Journal of Wildlife Diseases	*Ornithorhynchus anatinus* (platypus)	Australia	Wild Free-Living	4	no	Ulcerative dermatitis	Skin ulceration, ulcers were rough, greyish, granulating surface. severe, sup-purating, caseating granulomatous dermatitis/ myositis associated with the presence of large round organisms.	Skin	NA	Algae - (*Mucor sp*. Identified - but suggestive of a *Prototheca sp*. infection)	NA
Severe ulceronecrotic dermatitis associated with mite infestation in the critically endangered amargosa vole (*Microtus californicus scirpensis*)	Foley, J.<br>Branston, T.<br>Woods, L.<br>Clifford, D.	2013	Journal of Parasitology	*Microtus californicus scirpensis* (Amargosa vole)	United States of America	Wild Free-Living	60	yes	Ulceronecrotic dermatitis	Lesions, which included alopecia, swelling, marginal necrosis, and ulceration, as well as scarring, scabbing, and loss of pinna mass, parakeratotic hyperkeratosis and acanthosis with diffuse neutrophilic exocytosis and dense necrotic granulocytes in the epidermis and superficial dermis associated with focal erosion and ulceration.	Deformities on ears (pinnae) and chiggers on the ears and genitalia	Mite - (larval trombiculid in the genus *Neotrombicula*)	NA	NA
Sheep-associated malignant catarrhal fever–like skin disease in a free-ranging bighorn sheep (*Ovis canadensis*), Alberta, Canada	Slater, O. M.<br>Peters-Kennedy, J.<br>Lejeune, M.<br>Gummer, D.<br>Macbeth, B.<br>Warren, A.<br>Joseph, T.<br>Li, H.<br>Cunha, C. W.<br>Duignan, P. J.	2017	Journal of Wildlife Diseases	*Ovis canadensis* (bighorn sheep)	Canada	Wild Free-living	1	no	dermatitis	alopecia with hyperpigmentation and crusting, skin lesions,	face, medial surfaces of the pinnae, dorsal trunk, distal limbs, perineal area, and tail.	Virus - (Gammaherpesvirinae subfamily)	NA	Alberta, bighorn sheep, Canada, dermatitis, malignant catarrhal fever, ovine herpesvirus-2, Ovis canadensis, skin
A short review of the diseases of rhinoceros skin with case reports on an exudative dermatitis of the white rhinoceros (*Ceratotherium simum*)	Jones, DM<br>Thomsett, LR	1972	VerhBer. Erkrank. Zootier.	*Ceratotherium simum* (white rhinoceros)	England	Wild Captive	2	no	exudative dermatitis	superficial abrasions and lacerations of the skin, dermal erosions, erupting pustles, exudate, ulcers and pus	shoulders, thorax, neck, body surface,	NA	NA	NA
Skin-lesions in free-ranging black rhinoceroses (*Diceros-bicornis*) in Zimbabwe	Kock, N.<br>Kock, M. D.	1990	Journal of Zoo and Wildlife Medicine	*Diceros bicornis* (black rhinoceros)	Zimbabwe	Wild Free-Living	51	no	Ulcerative dermatitis	Lesions were ulcerated, exudative and crusty ulcerated, exudative, crusty, and erythematous and tended to be large (usually >5 cm wide)	Skin of the ventral neck	NA	Nematodes	Black rhinoceros, Diceros bicornis, filarial dermatitis
Spontaneous dermatophilosis in twin white-tailed deer fawns	Roscoe, Douglas E<br>Lund, Robert C<br>Gordon, Morris A<br>Salkin, Ira F	1975	Journal of wildlife diseases	*Odocoileus virginianus* (white-tailed deer)	United States of America	Wild Free-Living	2	no	Exudative dermatitis	Papular, darkly pigmented scabs, crusts, exudative	Muzzle, lips, ears	Bacteria - (*Dermatophilus congolensis*)	NA	NA
Spontaneous Pathology of the Gray Short-Tailed Opossum (*Monodelphis domestica*)	Hubbard, G. B.<br>Mahaney, M. C.<br>Gleiser, C. A.<br>Taylor, D. E.<br>Vandeberg, J. L.	1997	Laboratory Animal Science	*Monodelphis domestica* (gray short-tailed opossum)	United States of America	Wild Laboratory	10	no	Dermatitis	Gross lesions, crusts	Skin, ears	NA	NA	NA
Spontaneous poxviral dermatitis and keratoconjunctivitis in free-ranging mule deer (*Odocoileus hemionus*) in Wyoming	Williams, Es<br>Becerra, Vm<br>Thome, Et<br>Graham, Tj<br>Owens, Mj<br>Nunamaker, Ce	1985	Journal of wildlife diseases	*Odocoileus hemionus* (mule deer)	United States of America	Wild Free-Living	1	no	Ulcerative dermatitis	On gross examination there was severe mucopurulent bilateral keratoconjunctivitis with central corneal ulceration. The skin was crusty. Circular ulcers beneath tongue	Eyes, lips, muzzle and chin, hind feet	Virus - (parapoxvirus)	NA	NA
Squirrelpox virus: assessing prevalence, transmission and environmental degradation	Collins, Lisa M<br>Warnock, Neil D<br>Tosh, David G<br>McInnes, Colin<br>Everest, David<br>Montgomery, W Ian<br>Scantlebury, Mike<br>Marks, Nikki<br>Dick, Jaimie TA<br>Reid, Neil	2014	Plos One	*Sciurus vulgaris* (red squirrels)	Northern Ireland	Wild Free-living	1	yes	dermatitis	thick scabs and severe dermatitis.	lip	Virus - (squirrelpox)	NA	NA
Staphylococcic dermatitis in mink	Crandell, RA<br>Huttenhauer, GA<br>Casey, HW	1971	Journal of the American Veterinary Medical Association	*Neovison vison* (Mink)	United States of America	Wild Laboratory	1	no	dermatitis	lesions, elevated, smooth, yellow papules, crusty exudative, suppuratuive inflmation	head, neck, perineal area	Bacteria - (*Staphylococcus aureus*)	NA	NA
Staphylococcus intermedius Dermatitis in Denning New Jersey Black Bears (*Ursus americanus*)	Keeler, Shamus P<br>Burguess, Kelcey I<br>Lemaster, Heather<br>Huffman, Jane E	2012	Journal of the Pennsylvania Academy of Science	*Ursus americanus* (American black bear)	United States of America	Wild Free-living	4	no	Dermatitis	hair loss, crustiness, scaling, and reddening of their skin	body	Bacteria - (*Staphylococcus intermedius*)	NA	NA
Staphylococcus spp., Streptococcus canis, and Arcanobacterium phocae of healthy Canadian farmed mink and mink with pododermatitis	Chalmers, Gabhan<br>McLean, John<br>Hunter, D. Bruce<br>Brash, Marina<br>Slavic, Durda<br>Pearl, David L.<br>Boerlin, Patrick	2015	Canadian journal of veterinary research	*Neovison vison* (Mink)	Canada	Wild Captive	24	no	Pododermatitis	chronic hyperkeratotic purulent plantar dermatitis, suppurative pododermatitis, lesions	feet	NA	NA	NA
Steroidal saponin toxicity in eastern grey kangaroos (*Macropus giganteus*): A novel clinicopathologic presentation of hepatogenous photosensitization	Steventon, C. A.<br>Raidal, S. R.<br>Quinn, J. C.<br>Peters, A.	2018	Journal of Wildlife Diseases	*Macropus giganteus* (eastern grey kangaroo)	Australia	Wild Free-living	10	no	Dermatitis	skin lesions, excoriations, thickening, and scab formation, moderate to severe necrotizing or ulcerative lesions	skin	Plant - (*Panicum spp*.)	NA	Australia, blindness, hepatic photosensitization, macropod, marsupial, Panicum, plant poisoning, saponins.
Streptococcus didelphis sp nov., a streptococcus with marked catalase activity isolated from opossums (*Didelphis virginiana*) with suppurative dermatitis and liver fibrosis	Rurangirwa, F. R.<br>Teitzel, C. A.<br>Cui, J.<br>French, D. M.<br>Mcdonough, P. L.<br>Besser, T.	2000	International Journal of Systematic and Evolutionary Microbiology	*Didelphis virginiana* (opossum)	United States of America	Wild Captive	5	no	Suppurative dermatitis	Skin from five of the opossums was evaluated histologically. cutaneous lesions included suppurative, necrotizing dermatitis (3/5 animals) with extensive serocellular crusts (1/5) and necrotizing cellulitis (2/5) or dermal fibrosis (1/5).	Skin, flank, scrotum, axilla, tail, limbs	NA	Bacteria - (*Streptococcus spp; Streptococcus didelphis*)	NA
Subepidermal vesiculobullous filarial dermatitis in free-ranging American badgers (*Taxidea taxus*)	O'Toole, D<br>Williams, Es<br>Welch, V<br>Nunamaker, Ce<br>Lynn, C	1993	Veterinary pathology	*Taxidea taxus* (American badger)	United States of America	Wild Free-Living	26	no	Ulcerative dermatitis	Acute/cutaneous/chronic lesions, ulcerative superficial granulomatus dermatitis, necrosis	Inguinal region skin, medial thighs, abdomen	Nematodes - (*Filaria taxideae*)	NA	American badgers; Filaria taxideae; filariasis; lymphangitis; Taxidea taxus; vesiculobullous skin diseases.
Successful cyclosporine treatment for atopic dermatitis in a rhesus macaque (*Macaca mulatta*)	Ovadia, Shira<br>Wilson, Steven R<br>Zeiss, Caroline J	2005	Comparative Medicine	*Macaca mulatta* (Rhesus macaques)	United States of America	Wild Laboratory	1	no	Atopic dermatitis	Pruritus, erythema, alopecia, scaling, exfoliation, and lichenification, lesions	Ventrum, mandible and neck, axilla and inguinal regions, distal extremities, and interdigital regions	NA	Allergy dermatitis and generalized immune abnormalities.	NA
Successful treatment of generalized demodicosis in red-handed tamarins (*Saguinus midas*) using a single administration of oral fluralaner	Churgin, Sarah M.<br>Lee, Foo Khong<br>Groenvold, Kristin<br>Kovi, Ramesh C.<br>Cheung, Kam Yan<br>Martelli, Paolo R.<br>Zoo, Cert.	2018	Journal of Zoo and Wildlife Medicine	*Saguinus midas* (tamarins)	China	Wild Captive	2	no	dermatitis	skin masses, pyogranulomatous dermatitis, lesions, raised, plaque-like lesions, granulomatous dermatitis	eye, face, anterior surfaces of the forelimbs	Mite - (*Demodex sp*.)	NA	Acariasis, demodicosis, fluralaner, red-handed tamarin, Saguinus midas.

Superficial necrolytic dermatitis associated with hepatic lipidosis in a red fox (*Vulpes vulpes*)	Van Poucke, S.<br>Rest, J. R.	2005	Veterinary Record	*Vulpes vulpes* (red fox)	England	Wild Captive	1	no	dermatitis	superficial necrolytic dermatitis, hyperkeratosis, ulceration, erosions, crusts and alopecia	footpads, carpi, hocks, elbows	NA	NA	NA
A survey of urinary tract disease in New South Wales koalas	Canfield, P. J.	1989	Australian Veterinary Journal	*Phascolarctos cinereus* (koala)	Australia	Wild Free-Living	3	no	Dermatitis	NA	Skin	NA	Compromised immune system due to cystitis	NA
Suspected dermatophilosis in an adult orangutan (*Pongo pygmaeus pygmaeus*)	Brack, Manfred<br>Hochleithner, Claudia<br>Hochleithner, Manfred<br>Zenker, Wolfgang	1997	Journal of Zoo and Wildlife Medicine	*Pongo pygmaeus pygmaeus* (Northwest Bornean Orangutan)	Austria	Wild Captive	1	no	Dermatitis	Pruritic, vesicular skin disease, crusts	Extremities, trunk, and face	Bacteria - (*Dermatophilus congolensis*)	NA	Skin disease, orangutan, Dermatophilus congolensis
Suspected Pyrrolizidine Alkaloid Hepatotoxicosis in Wild Southern Hairy-Nosed Wombats (*Lasiorhinus latifrons*)	Woolford, Lucy<br>Fletcher, Mary T.<br>Boardman, Wayne S. J.	2014	Journal of Agricultural and Food Chemistry	*Lasiorhinus latifrons* (Southern hairy-nosed wombat)	Australia	Wild Free-living	10	yes	Dermatitis	alopecia, dermatitis, necrosis, skin lesions, exudative necrosuppurative and hemorrhagic dermatitis with superficial vascular degeneration, thrombosis, and infarction.	body, head, eyelid, ears	Plant - (*Heliotropeum europaeum*)	NA	pyrrolizidine alkaloids, wombat, Lasiorhinus latifrons, hepatotoxic, Heliotropeum europaeum
Systematic assessment of the impact of adenovirus infection on a captive reintroduction project for red squirrels (*Sciurus vulgaris*)	Everest, DJ<br>Shuttleworth, CM<br>Grierson, SS<br>Duff, JP<br>Jackson, N<br>Litherland, P<br>Kenward, RE<br>Stidworthy, MF	2012	Veterinary Record	*Sciurus vulgaris* (red squirrels)	Wales	Wild Free-living	1	yes	dermatitis	severe staphylococcal dermatitis	facial and footpad	Bacteria - (*Staphylococcus sp*.)	NA	NA
Syphilis in European brown hares (*Lepus europaeus*)	Lumeij, JT	1996	Veterinary quarterly	*Lepus europaeus* (European brown hares)	Netherlands	Wild Free-living	2	no	dermatitis	skin lesions, dermatitis,	lips, prepuce	Bacteria - (*Treponema sp*.)	NA	NA
Tacrolimus ointment: a novel and effective topical treatment of localized atopic dermatitis in a rhesus macaque (*Macaca mulatta*)	Torreilles, Stéphanie L<br>Luong, Richard H<br>Felt, Stephen A<br>Mcclure, Diane E	2009	Journal of the American Association for Laboratory Animal Science	*Macaca mulatta* (Rhesus macaques)	United States of America	Wild Laboratory	1	no	Atopic dermatitis	Focal, exudative, inflamed, erythematous skin lesion, raised lesions, alopecia, scaling, lichenification, and exfoliation	Mandible	NA	Allergy dermatitis (atopic dermatitis)	NA
Thadeua greeni–associated dermatitis in an eastern bettong (*Bettongia gaimardi*)	Portas, Timothy J<br>Taylor, David<br>Spratt, David M	2015	Journal of Zoo and Wildlife Medicine	*Bettongia gaimardi* (bettong)	Australia	Wild Free-Living	1	no	Dermatitis	Extensive alopecia, scaling, crusting and dermatitis, chronic proliferative and hyperkeratotic perivascular dermatitis with intralesional mites	Ventral and lateral aspects of the neck and thorax.	Mite - (*Thadeua greeni*)	NA	Bettongia gaimardi; Thadeua greeni; dermatitis; eastern bettong; mite
Therapeutic management of bacterial dermatitis in captive Asian elephant (*Elephas maximus)*	Gogoi, P<br>Phukan, A<br>Sharma, RK<br>Mahato, G<br>Hazarika, RA<br>D Boro, PK	2017	Journal of Entpmology and Zoology Studies	*Elephas maximus* (Asian Elephant)	India	Wild captive	21	no	Dermatitis	marked pyogenic lesions	trunk, neck, abdomen, tail, back, thigh, leg and in the foot<br>regions.	Bacteria - (*Staphylococcus* sp.)	NA	Captive Asian elephants, Bacterial dermatitis, *S. aureus, S.intermedius,S.hyicus, S. saprophyticus, S. caseolyticus,* and *Amoxicillin/Clavulanic acid*
Trauma found to be a significant cause of death in a pathological investigation of bent-winged bats (*Miniopterus orianae*)	Holz, Peter H.<br>Stent, Andrew<br>Lumsden, Linda F.<br>Hufschmid, Jasmin	2020	Journal of Zoo and Wildlife Medicine	Miniopterus orianae oceanensis (eastern bent-wing bat)	Australia	Wild Free-living	1	yes	dermatitis	hyperkeratotic lesions,	dorsal surface of both wings	Mite - (*Notoedres muris*)	NA	Eastern bent-winged bat, Miniopterus orianae bassanii, Miniopterus orianae oceanensis, mite, Notoedres muris, southern bent-winged bat
Treatment of chronic herpesviral dermatitis in a captive cheetah (*Acinonyx jubatus*) in Namibia	Flacke, Gabriella L<br>Schmidt-Küntzel, Anne<br>Marker, Laurie	2015	Journal of Zoo and Wildlife Medicine	*Acinoyx jubatus* (cheetah)	Namibia	Wild Captive	1	no	Feline chronic herpesviral dermatitis	Cutaneous lesions consisting of alopecia, erythema, ulceration and crusting	Left fore and hind limbs	Virus - (Feline herpesvirus-1 (FHV-1))	NA	*Acinonyx jubatus;* cheetah; cryotherapy; famciclovir; feline herpesvirus-1 (FHV-1); ulcerative dermatitis
Treatment of mange caused by Caparinia tripilis in native Korean wild hedgehogs (*Erinaceus amurensis*): a case report	Eo, K. Y.<br>Kwak, D.<br>Kwon, O. D.	2015	Veterinarni Medicina	*Erinaceus amurensis* (Korean hedgehog)	South Korea	Wild Captive	1	yes	dermatitis	pruritic dermatitis, skin lesions	outer ears and upper chest	Mite - (*Caparinia tripilis*)	NA	capariniosis; mange; Erinaceus amurensis; native Korean wild hedgehogs; treatment
Treatment of vasculitis and dermatitis in a 59-yr-old Nile hippopotamus (*Hippopotamus amphibius*)	Spriggs, Maria<br>Reeder, Chris	2012	Journal of Zoo and Wildlife Medicine	*Hippopotamus amphibius* (Nile hippopotamus)	United States of America	Wild Captive	1	no	Dermatitis	Ulcers, inflamtion, focal erosions skin lesions were hyperemic, and several had purulent and malodorous discharge	Skin folds, vulva, and tongue, axillae, groin, perivulvar region, and flanks	NA	Bacteria - (*Morganella morgannii*, and *Enterococcus sp*)	Dermatitis, hippopotamus, *Hippopotamus amphibius*, skin, *Streptococcus, vasculitis*.
Treatment success in three Andean bears (*Tremarctos ornatus*) with Alopecia syndrome using oclacitinib maleate (Apoquel®)	Drake, Gabby J<br>Nuttall, Tim<br>López, Javier<br>Magnone, William<br>Leclerc, Antoine<br>Potier, Romain<br>Lécu, Alexis<br>Guézénec, Maëlle<br>Kolter, Lydia<br>Nicolau, Amélie	2017	Journal of Zoo and Wildlife Medicine	*Tremarctos ornatus* (Andean bear)	France, Italy, England	Wild Captive	3	yes	Atopic dermatitis	Alopecia, pruritus, hyperkeratosis, scaling, dry, crusted	Back and flanks, periorbital,<br>and medial thigh skin	NA	Allergy dermatitis (atopic dermatitis), Fungus (*Malassezia spp.*)	Alopecia, Andean bear, oclacitinib maleate, pruritus, spectacled bear, Tremarctos ornatus.
Treponemal infections in hares in The Netherlands	Lumeij, J. T.<br>de Koning, J.<br>Bosma, R. B.<br>van der Sluis, J. J.<br>Schellekens, J. F.	1994	Journal of clinical microbiology	*Lepus europaeus* (European brown hares)	Netherlands	Wild Free-living	2	no	Dermatitis	skin lesions, ulcers, crusts, dermatitis	oral skin and the prepuce, anogenital area, scrotal	Bacteria - (*Treponema sp.*)	NA	NA
Treponeme-associated hoof disease of free-ranging elk (*Cervus elaphus*) in Southwestern Washington State, USA	Han, Sushan<br>Mansfield, Kristin G<br>Bradway, Dan S<br>Besser, Thomas E<br>Read, Deryck H<br>Haldorson, Gary J<br>Alt, David P<br>Wilson-Welder, Jennifer H	2019	Veterinary pathology	*Cervus elaphus* (red deer)	United States of America	Wild Free-Living	2	no	Dermatitis	Cutaneous foot lesion with interdigital ulcerative dermatitis. Suppurative and erosive interdigital dermatitis with chronic acanthosis. necrotizing dermatitis	Foot	NA	Dermatitis was associated with Treponema sp. Bacteria, in combination with a diversity of aerobic and anaerobic bacteria.	Cervus elaphus, digital dermatitis, elk, sole ulcers, spirochete, Treponema, treponeme-associated hoof disease
Trombiculiasis in the Florida black bear	Cunningham, Mark W<br>Phillips, Lynette A<br>Welbourn, Cal	2001	Journal of Wildlife Diseases	*Ursus americanus floridanus* (Florida black bear)	United States of America	Wild Free-Living	2	no	Dermatitis	Pustular cutaneous lesions, crusting and scaling	Ventral abdomen, inguinal, and axillary regions, entire body	Mite - (*Trombiculidae spp. Eutrombicula splendens*)	NA	Black bear, chigger, dermatitis, Eutrombicula splendens, Trombiculidae, trombiculiasis, Ursus americanus floridanus
Trombidiosis-induced dermatitis in white-tailed deer (*Odocoileus virginianus*)	Little, S. E.<br>Carmichael, K. P.<br>Rakich, P. M.	1997	Veterinary Pathology	*Odocoileus virginianus* (white-tailed deer)	United States of America	Wild Free-Living	6	no	Plasmacytic dermatitis	Orange granular material. Serous exudate, crusting, and alopecia. severe chronic diffuse plasmacytic dermatitis with intralesional larval trombiculid mites	Eyes and mouth	Mite - (*Trombiculidae spp.*)	NA	chiggers, dermatitis, odocoileus virginianus, trombiculidae, white-tailed deer
Use of oral fluralaner (Bravecto) to successfully manage adult-onset generalised demodicosis in a silver fox (*Vulpes vulpes*)	Burke, A.<br>Kottwitz, J.<br>Wang, C.<br>White, A.	2019	Veterinary Record Case Reports	*Vulpes vulpes* (red fox)	United States of America	Wild Captive	1	no	Dermatitis	alopecia, crusts, nodules, pustules, crusts	dorsal muzzle, distal extremities, limbs	Mite - (*Demodex canis*)	NA	NA
Vaccine-induced canine distemper in European mink, *Mustela lutreola*	SutherlandSmith, M. R.<br>Rideout, B. A.<br>Mikolon, A. B.<br>Appel, M. J. G.<br>Morris, P. J.<br>Shima, A. L.<br>Janssen, D. J.	1997	Journal of Zoo and Wildlife Medicine	*Mustela lutreola* (European mink)	United States of America	Wild Captive	4	yes	Dermatitis	multifocal papulopustular dermatitis, skin lesions, necrotizing dermatitis	axillary areas	Virus - (Canine distemper virus)	NA	Vaccine-induced canine distemper, avian-origin canine distemper vaccine, Mustela lutreola,
Vesicular and ulcerative dermatopathy resembling superficial necrolytic dermatitis in captive black rhinoceroses (*Diceros bicornis*)	Munson, L<br>Koehler, J Wilkinson<br>Wilkinson, Je<br>Miller, Re	1998	Veterinary Pathology	*Diceros bicornis* (black rhinoceros)	United States of America	Wild Captive	40	yes	Superficial necrolytic dermatitis	Lesions as vesicles, bullae, or ulcers, ulceration, parakeratos	Pressure points, coronary bands, tips of the ears and tail, and along the lateral body wall<br>and dorsum, lips	NA	The high prevalence<br>of this skin disease in captive black rhinoceroses under many circumstances suggests that their epidermis is acutely sensitive to any disruption of metabolic homeostasis	Black rhinoceros; eosinophilic granulomas; hepatocutaneous syndrome; necrolytic migratory erythema; skin; superficial necrolytic dermatitis; ulcerative skin disease

West Nile virus infection in free-ranging squirrels in Illinois	Heinz-Taheny, Kathleen M<br>Andrews, John J<br>Kinsel, Michael J<br>Pessier, Allan P<br>Pinkerton, Marie E<br>Lemberger, Karin Y<br>Novak, Robert J<br>Dizikes, George J<br>Edwards, Eric<br>Komar, Nicholas	2004	Journal of veterinary diagnostic investigation	*Sciurus carolinensis* (gray squirrel)	United States of America	Wild Captive	1	no	dermatitis	lesion, mild proliferative dermatitis	NA	NA	NA	NA
Winter tick (*Dermacentor albipictu* s)–associated dermatitis in a wild elk *(Cervus canadensis)* in Pennsylvania, USA	Calvente, E.<br>Chinnici, N.<br>Brown, J.<br>Banfield, J. E.<br>Brooks, J. W.<br>Yabsley, M. J.	2020	Journal of Wildlife Diseases	*Cervus canadensis* (elk)	United States of America	Wild Free-living	1	yes	Ulcerative dermatitis	Severe chronic-active dermatitis with hyperkeratosis and crust formation, alopecia	neck, dorsum region	Tick - (*Dermacentor albipictus*)	NA	NA
Zinc-responsive dermatosis in a red wolf (*Canis rufus*)	Kearns, Karen<br>Sleeman, Jonathan<br>Frank, Linda<br>Munson, Linda	2000	Journal of Zoo and Wildlife Medicine	*Canis rufus* (red wolf)	United States of America	Wild Captive	1	yes	Dermatitis	Hyperkeratosis, inflamed skin, ulcers, crusts	Limbs, feet, footpads, digits	Environmental - (*Zinc deficiency*)	NA	Canis rufus, dermatosis, parakeratosis, nutritional dermatosis, red wolf, zinc

Table S3. All individually reported dermatitis cases for each order. These orders were included in further analysis but since each order had 10 or less dermatitis diagnoses and fewer than five species within the grouping, their results are displayed here rather than in-text. The number of cases for each species, split into captivity status (wild free-living (WFL), wild captive (WC), and wild laboratory (WL)) for the corresponding cause.

Order	Species	Cause	Cases		
			**WFL**	**WC**	**WL**
Didelphimorphia					
	Didelphis virginiana	Unknown		1	
	Monodelphis domestica	Unknown			1
Eulipotyphla					
	Erinaceus europaeus	Bacteria	1		
	Atelerix albiventris	Mite		2	
	Erinaceus albiventris	Unknown		1	
	Erinaceus amurensis	Mite		1	
Lagomorpha					
	Lepus timidus	Virus	1		
	Pentalagus furnessi	Mite	1		
	Sylvilagus floridanus	Bacteria	1		
	Lepus europaeus	Bacteria	2		
Perissodactyla					
	Ceratotherium simum	Fungus		1	
		Unknown		2	
	Diceros bicornis	Nematode	1		
		Unknown	1	3	
	Rhinoceros unicornis	Fungus		1	
		Unknown		1	
Chiroptera					
	Eptesicus fuscus	Unknown	1		
	Miniopterus orianae oceanensis	Mite	1		
	Vespertilio murinus	Fungus	1		
	Vespertilionidae (family)	Unknown	1		
Other					
	Elephas maximus	Unknown		1	
		Bacteria		1	
	Ornithorhynchus anatinus	Fungus	1		
		Unknown	1		
	Procavia capensis	Mite		1	
	Zaedyus pichiy	Unknown	1	1	

Table S4. Etiological agents responsible for the causes of dermatitis across terrestrial and semi-aquatic mammalian wildlife species. There were over 60 causal agents of dermatitis for the 108 wildlife species, some agents were only identified to the nearest family or genus, or in some cases were reported as an unknown agent (e.g., 'unknown nematode'). The causes of dermatitis, the etiological agents, and the total number of wild mammal species for each are shown below.

**Bacteria**	Species
Dermatophilus congolensis	18
Chromobacterium violaceum	2
Dichelobacter nodosus	1
Staphylococcus sp. (genus)	7
Arcanobacterium sp. (genus)	2
Mycobacterium lepromatosis	1
Streptomyces sp. (genus)	1
Treponema sp. (genus)	2

**Nematode**	Species
Filaria taxideae	3
Unknown nematode	2
Pelodera strongyloides	1

**Mite**	Species
Demodex sp. (genus)	8
Notoedres sp. (genus)	8
Sarcoptes scabiei	7
Trombiculidae (family)	3
Ornithonyssus bacoti	2
Thadeua serrata	2
Psoroptes sp. (genus)	2
Caparinia tripilis	2
Chorioptes sp. (genus)	2
Straelensia cynotis	1
Leptotrombidium miyajimai	1
Neotrombicula sp. (genus)	1
Odontacarus adelaideae	1
Psorergates cercopitheci	1
Psorobia sp. (genus)	1
Thadeua greeni	1
Ursicoptes americanus	1

**Fungus**	Species
Malassezia sp. (genus)	6
Trichophyton mentagrophytes	4
Candida sp. (genus)	4
Fusarium sp. (genus)	3
Yarrowia (Candida) lipolytica	3
Unknown fungus	1
Alternaria sp. (genus)	1
Mucor amphibiorum	1
Curvularia sp. (genus)	1
Pityrosporum pachydermatis	1
Cladosporium sp. And *Rhodotorula sp.*	1
Unknown dermatophytosis	1
Phoma cava	1
Lodderomyces elongisporus	1

**Tick**	Species
Ixodes sp. (genus)	3
Haemaphysalis bancrofti	2
Amblyomma maculatum and *A. americanum*	1
Amblyomrrw triguttatum	1
Dermacentor albipictus	1

**Virus**	Species
Parapoxvirus (genus)	13
Poxviridae (family)	2
Felid alphaherpesvirus 1	1
Squirrelpox virus	1
Gammaherpesvirinae subfamily	1
Canine morbillivirus	1
Cervidpoxvirus (genus)	1

**Diptera**	Species
Lipopte cervi	2
Dermatobia hominis	1

**Bacteria & Fungus**	Species
Bacteria (*Arcanobacterium haemolyticum, Staphylococcus sp.*, *Streptococcus sp.*) & Fungus (unknown)	1
Bacteria (*Dermatophilus congolensis*) & Fungus (*Alternaria alternata*)	1

**Louse**	Species
Neohaematopinus sciuri	1
Heterodoxus ualabati	1

**Other**	Species
Apicomplexan – *Besnoitia sp.* (genus)	2
Fleas – *Echidnophaga sp.* (genus)	2
Mineral deficiency	1
Ectoparasites - mites, louse, or deer ked	1
Protozoa - *Neospora caninum*	1
Mite (*Notoedres cati*) & Fungus (*Malassezia sp.*)	1
Mite (*Sarcoptes scabiei*) & Nematode (*Pelodera strongyloides*)	1

**Plant**	Species
Panicum sp. (genus)	1
Heliotropeum europaeum	1
Lantanan camara	1
Bidens laevis	1

Table S5. Each reported IUCN (2021) threatened species dermatitis case: the dermatitis cause is listed; whether any additional concurrent disease or disorders were identified in the individual(s); whether the dermatitis cause was treated; and whether the treatment successfully resolved the dermatitis, unsuccessful treatments either resolved in euthanasia or death of the individual(s). Treatment of dermatitis was attempted in 26 cases (58%), which consisted of medical intervention for the cure of the cause of dermatitis (e.g., antibiotics; ivermectin), or for mitigation of clinical signs (e.g., removal of lesions) if the cause was chronic (e.g., Feline Herpes Virus in Cheetahs). Treatment was deemed successful in most instances (n = 17), unsuccessful in six cases, and not reported in three (NA). Treatment was not attempted in 19 cases (42%), because these were either: free living animals (5), spontaneously recovered (3), died from no treatment (1), were presented dead or euthanized on arrival for necropsy (9), or there was insufficient data (1). Notes for some cases are provided due to the complexity of treatment success in species.

**IUCN (2021) Threatened Species cases**	**Dermatitis cause**	**Total individuals**	**Concurrent disease//disorder identified?**	**Treatment**	**Successful**	**Note**
Canis rufus (Red Wolf)	Mineral Deficiency	1	No	Yes	Yes	Recovered
Dasyprocta mexicana (Mexican Agouti)	Fleas	3	No	Yes	Yes	Recovered
Diceros bicornis (Black Rhinoceros)	Unknown	40	Yes	Yes	Yes/No	No: death from concurrent diseases. Treatment success was variable, and some lesions resolved without treatment
Diceros bicornis (Black Rhinoceros)	Unknown	8	Yes	Yes	Yes/No	3 treated: 2 died, 1 recovered
Diceros bicornis (Black Rhinoceros)	Unknown	51	No	NA	NA	NA: wild free-living
Diceros bicornis (Black Rhinoceros)	Unknown	1	Yes	Yes	No	Died
Diceros bicornis (Black Rhinoceros)	Nematode	1	No	No	NA	NA: wild free-living
Mustela lutreola (European Mink)	Virus	4	No	Yes	No	No: 1 died during operation, all 3 were treated died
Pongo pygmaeus pygmaeus (Northwest Bornean Orangutan)	Bacteria	1	No	Yes	Yes	Recovered
Ailurus fulgens (Red Panda)	Nematode	1	No	No	No	Death
Ailurus fulgens (Red Panda)	Bacteria	1	No	Yes	No	Death
Elephas maximus (Asian Elephant)	Unknown	1	No	Yes	yes	-
Elephas maximus (Asian Elephant)	Bacteria	21	No	Yes	Yes	-
Pentalagus furnessi (Amami Rabbit)	Mite	1	No	NA	NA	Found Dead
Petrogale persephone (Proserpine Rock Wallaby)	Mite	6	No	NA	NA	Found Dead
Symphalangus syndactylus (Siamang)	Mite	2	No	Yes	Yes	Recovered

Acinonyx jubatus (Cheetah)	Virus	3	No	Yes	No	Chronic disease and treatment was not successful
Acinonyx jubatus (Cheetah)	Virus	20	Yes	Yes	No	Chronic disease and treatment were minimally responsive
Acinonyx jubatus (Cheetah)	Virus	50	No	Yes	NA	Chronic disease
Acinonyx jubatus (Cheetah)	Virus	21	No	Yes	NA	Insufficient data
Acinonyx jubatus (Cheetah)	Virus	1	Yes	Yes	No	Euthanized
Alouatta palliata (Mantled Howler Monkey)	Bacteria	1	No	NA	NA	Euthanized on arrival
Budorcas taxicolor tibetana (Sichuan Takin)	Virus	2	No	No	Yes	Resolved spontaneously
Hippopotamus amphibius (Nile Hippopotamus)	Unknown	1	No	Yes	Yes	-
Hippopotamus amphibius (Nile Hippopotamus)	Unknown	3	No	Yes	Yes	-
Hippopotamus amphibius (Nile Hippopotamus)	Unknown	3	No	NA	NA	Insufficient data
Lagothrix lagotricha (Woolly Monkey)	Bacteria	1	No	Yes	Yes	-
Macaca fascicularis (Crab-eating Macaque)	Unknown	1	No	No	Yes	Lesions resolved spontaneously
Macaca fascicularis (Crab-eating Macaque)	Bacteria	2	No	No	Yes	Resolved spontaneously
Petrogale penicillata (Brush-tailed Rock-wallaby)	Tick	88	No	NA	NA	NA: wild free-living
Phascolarctos cinereus (Koala)	Unknown	3	No	NA	NA	Dead on arrival
Rangifer tarandus (Caribou)	Virus	1	Yes	NA	NA	Dead on arrival
Rangifer tarandus (Caribou)	Virus	14	No	NA	NA	NA: experiment, result was euthanasia
Rangifer tarandus (Caribou)	Apicomplexan	23	Yes	NA	NA	Euthanized
Rangifer tarandus (Caribou)	Apicomplexan	4	No	NA	NA	Euthanized

Rhinoceros unicornis (Indian Rhinoceros)	Fungus	2	No	Yes	Yes	-
Rhinoceros unicornis (Indian Rhinoceros)	Unknown	1	Yes	No	NA	Died due to concurrent disease or euthanized
Rusa unicolor (Sambar)	Diptera	8	No	Yes	Yes	-
Tremarctos ornatus (Andean Bear)	Unknown	3	No	Yes	Yes	-
Tremarctos ornatus (Andean Bear)	Unknown	96	Yes	NA	NA	Insufficient data
Tremarctos ornatus (Andean Bear)	Unknown	20	No	Yes	NA	NA: Some treatment reported, but insufficient data on success, no deaths reported
Ursus maritimus (Polar Bear)	Bacteria	1	No	Yes	Yes	-
Ursus maritimus (Polar Bear)	Bacteria	2	No	Yes	Yes	-
Ursus maritimus (Polar Bear)	Unknown	49	No	No	NA	NA: wild free-living; 12 spontaneously recovered
Ursus maritimus (Polar Bear)	Bacteria	2	No	Yes	Yes	-
